# Discovery of paradoxical genes: reevaluating the prognostic impact of overexpressed genes in cancer

**DOI:** 10.3389/fcell.2025.1525345

**Published:** 2025-01-22

**Authors:** Dequan Liu, Lei Liu, Xiangyu Che, Guangzhen Wu

**Affiliations:** Department of Urology, The First Affiliated Hospital of Dalian Medical University, Dalian, China

**Keywords:** paradoxical genes, bioinformatics, tumor metabolism, discordant gene-protein abundance, tumor immune microenvironment, signaling pathway

## Abstract

Oncogenes are typically overexpressed in tumor tissues and often linked to poor prognosis. However, recent advancements in bioinformatics have revealed that many highly expressed genes in tumors are associated with better patient outcomes. These genes, which act as tumor suppressors, are referred to as “paradoxical genes.” Analyzing The Cancer Genome Atlas (TCGA) confirmed the widespread presence of paradoxical genes, and KEGG analysis revealed their role in regulating tumor metabolism. Mechanistically, discrepancies between gene and protein expression-affected by pre- and post-transcriptional modifications-may drive this phenomenon. Mechanisms like upstream open reading frames and alternative splicing contribute to these inconsistencies. Many paradoxical genes modulate the tumor immune microenvironment, exerting tumor-suppressive effects. Further analysis shows that the stage- and tumor-specific expression of these genes, along with their environmental sensitivity, influence their dual roles in various signaling pathways. These findings highlight the importance of paradoxical genes in resisting tumor progression and maintaining cellular homeostasis, offering new avenues for targeted cancer therapy.

## Graphical Abstract

**Figure:**
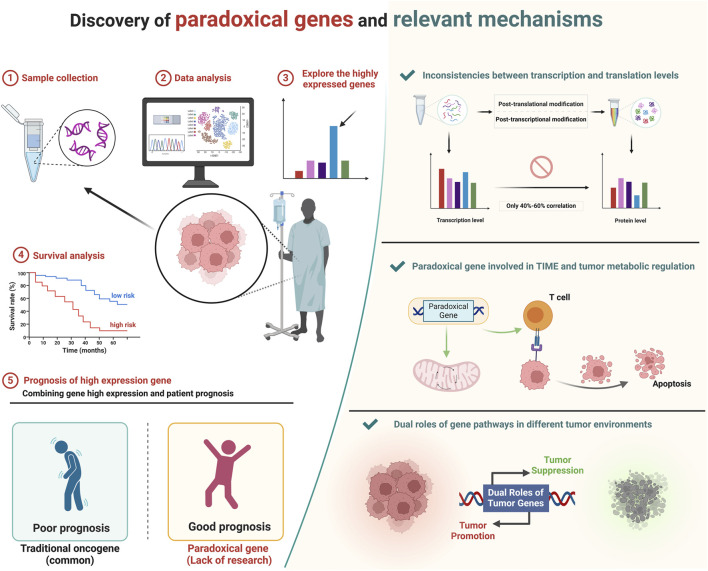
Typically, oncogenes show higher expression in tumors and are linked to poor prognoses. However, some highly expressed genes in tumors are associated with better outcomes and act as tumor suppressors, termed paradoxical genes. We explored these genes using bioinformatics, revealing their significant roles in regulating tumor metabolism and immune microenvironments, offering new insights for targeted cancer therapy. Created in BioRender. ZHAO, X. (2025) https://BioRender.com/b00e214.

## 1 Introduction

Traditionally, bioinformatics analyses often utilize the differential gene abundance between tumor and normal tissues to screen for target genes (Golub et al., 1999). Typically, genes with significantly higher abundance in tumors than normal tissues are classified as oncogenes and become focal points of research (Bishop, 1991; Croce, 2008). However, a new understanding has emerged with the proliferation of comprehensive databases such as TCGA. Researchers increasingly recognize that not all genes are highly expressed in tumors act as promoters of cancer (Cao et al., 2021; Yan et al., 2019; Boor et al., 2020; Martinez-Turtos et al., 2022). In fact, some genes show high abundance in tumor tissues but are associated with good patients prognosis (Cao et al., 2021; Yan et al., 2019; Boor et al., 2020; Martinez-Turtos et al., 2022). This finding challenges the traditional understanding of tumor biology. Although public databases provide evidence that genes which are highly expressed in tumor tissues and serve tumor suppressor roles are prevalent, they have not been broadly recognized or systematically analyzed by the scientific community. Our work pioneers the classification and definition of these genes as “paradoxical genes,” and it delves deeply into the reasons and context for the existence of these paradoxical genes.

The discrepancy between mRNA and protein levels may be a reason for the emergence of paradoxical genes (Vogel and Marcotte, 2012). This difference is mainly due to post-transcriptional and -translational modifications, which are crucial in the dynamic regulation of gene expression (Vogel and Marcotte, 2012). Post-transcriptional modifications include processes such as alternative splicing, enabling a single gene to generate multiple mRNA variants, thereby expanding the diversity of the proteome (Vogel and Marcotte, 2012; Wahl et al., 2009). The upstream open reading frames (uORFs) can significantly regulate the translation of the main open reading frame (ORF) (Barbosa et al., 2013; Johnstone et al., 2016). Concurrently, post-translational modifications such as phosphorylation and ubiquitination further diversify protein functions and regulation (Hershko and Ciechanover, 1998; Hunter, 2007). However, within the context of TCGA, the focus is solely on the measurement of mRNA abundance (Cancer Genome Atlas Research Network, 2008; Author anonymous, 2012; Weinstein et al., 2013). This is typically quantified using RNA sequencing data, which provides detailed information on the levels of mRNA present in a given sample (Cancer Genome Atlas Research Network, 2008; Author anonymous, 2012; Weinstein et al., 2013). Inconsistencies between gene and protein levels can potentially distort patient prognosis results, contributing to the emergence of paradoxical genes (Akbani et al., 2014).

The tumor immune microenvironment (TIME) is critical for suppressing tumor progression, through its complex network of immune cells, stromal cells, signaling molecules, and extracellular matrix components (Joyce and Fearon, 2015; Fridman et al., 2012; Quail and Joyce, 2013). This dynamic environment can promote or inhibit tumor growth, depending on the balance of pro- and anti-tumor factors (Schreiber et al., 2011). Key players include cytotoxic T lymphocytes (CTLs), natural killer (NK) cells, dendritic cells, B cells, proinflammatory cytokines, and chemokines (Schreiber et al., 2011; Smyth et al., 2002; Palucka and Banchereau, 2012; Nelson, 2010; Müller et al., 2009; Topalian et al., 2015). There is currently evidence that some Paradoxical genes are involved in regulating TIME and inhibiting tumor progression (Cao et al., 2021; Yan et al., 2019; Boor et al., 2020; Martinez-Turtos et al., 2022). The regulatory influence of paradoxical genes on TIME elucidates the mechanism underlying their tumor suppressor effects.

The expression of genes and pathways exhibits context-dependent effects, also known as context specificity, which refer to the phenomenon where the function or behavior of a gene varies depending on the specific context in which it operates (Feil and Fraga, 2012; Huang, 2009; Beyer et al., 2007). In the context of cancer, the role of a gene can vary significantly depending on factors such as the type of cancer, the tissue in which the tumor originates, and the stage of the cancer (Vogelstein and Kinzler, 2004; Hanahan and Weinberg, 2011; Stratton et al., 2009). For instance, genes associated with signaling pathways like TGFβ, NOTCH, and NF-κB demonstrate differential expression across various tumor tissues. The TGFβ pathway presents a dual role, acting as a tumor suppressor in early stages and a promoter in advanced stages, with the expression varying significantly in breast, pancreatic, and colorectal cancers (Massagué, 2008). Similarly, the NOTCH signaling pathway, critical for cell fate determination and differentiation, shows oncogenic as well as tumor-suppressive functions, depending on the cancer type, as observed in breast cancer (BRCA), T-cell acute lymphoblastic leukemia, and lung cancer (Stylianou et al., 2006; Weng et al., 2004; Kopan and Ilagan, 2009). Often, the NF-κB pathway, a key regulator of inflammation and immune responses, is dysregulated in BRCA, multiple myeloma, and colorectal cancer, where it facilitates cell proliferation, survival, and chronic inflammation (Karin, 2006; Annunziata et al., 2007; Greten et al., 2004). The specific roles and expression patterns of these similar pathways in different tumor environments are also one of the reasons for the widespread existence of paradoxical genes.

In this article, by exploring the mechanism of paradoxical genes formation, we seek to broaden the current understanding of tumor biology and provide new ideas for tumor treatment and research.

## 2 Paradoxical genes emerge as a key focus in bioinformatics research

When genes highly expressed in cancer compared to normal tissues, are identified through bioinformatics analyses, it often implies that these genes may act as drivers of oncogenesis, serve as diagnostic or prognostic biomarkers, or act as predictive markers for treatment response (Niu et al., 2022; Liu Y. et al., 2021). Indeed, the relationship between gene expression and cancer prognosis can be complex and sometimes counterintuitive (Hanahan and Weinberg, 2011). Contrary to what might be expected, high expression of certain genes in tumors can be associated with a more favorable prognosis (Hanahan and Weinberg, 2011). This paradoxical finding highlights the complexity of cancer biology, revealing that genes may play multifaceted roles in tumorigenesis and cancer progression (Hanahan and Weinberg, 2011). For instance, some genes highly expressed in tumors could participate in immune response activation, DNA repair mechanisms, or cellular differentiation processes, potentially inhibiting tumor growth or spread, thereby improving patient outcomes (Blagih et al., 2020; Williams and Schumacher, 2016; Kalluri and Weinberg, 2009). Researchers face a challenge of dissecting the dual roles that some genes, acting as oncogenes in certain contexts while serving protective or suppressive functions in others (Hanahan and Weinberg, 2011).

### 2.1 Paradoxical genes exhibit differential and uneven expression across various tumors

**FIGURE 1:**
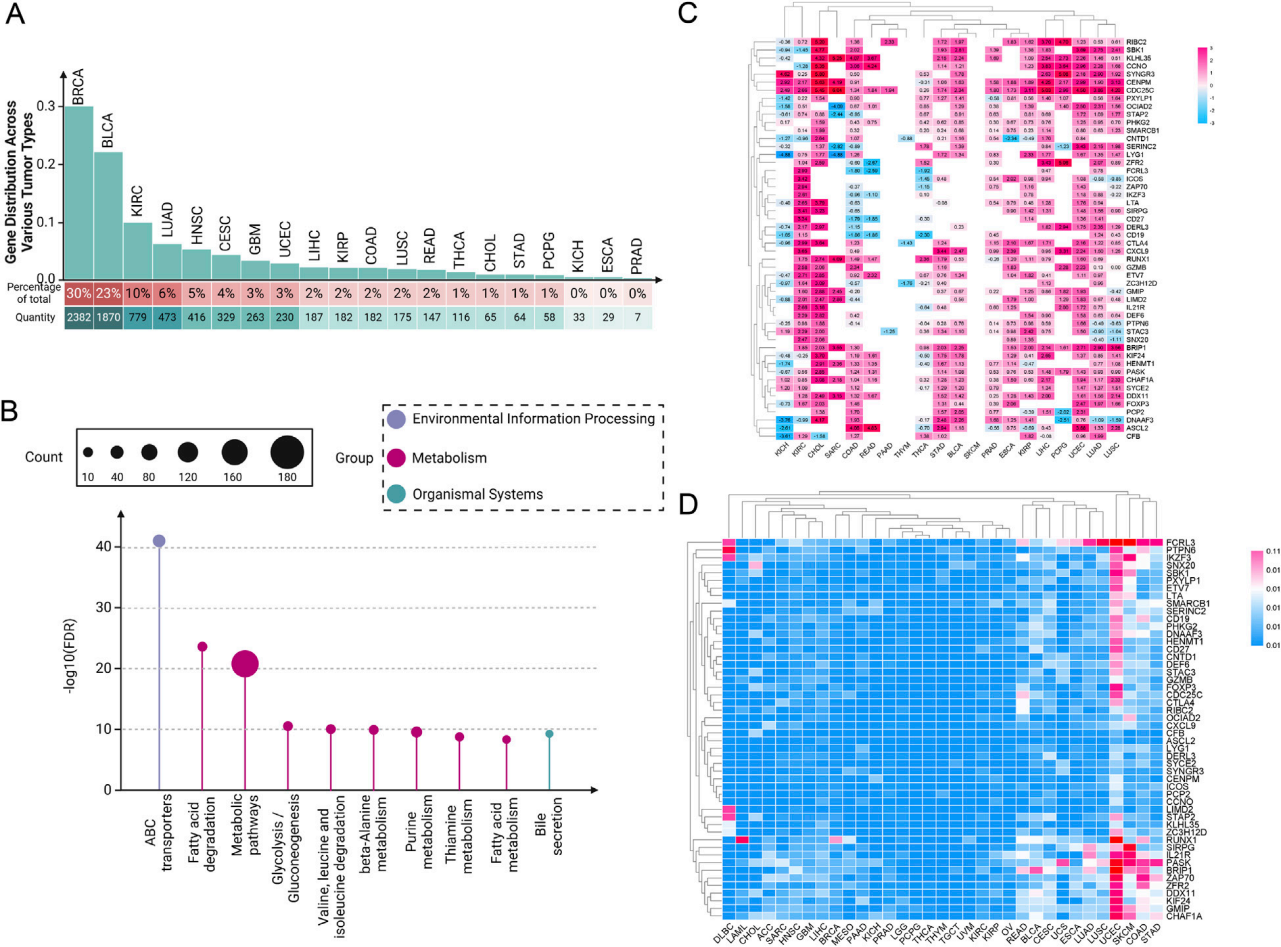
Pan-cancer analysis of paradoxical genes. **(A)** Expression of paradoxical genes in various tumors. The proportion of paradoxical genes in tumors is rounded to the nearest whole number; **(B)** KEGG analysis of 254 paradoxical genes; **(C)** Heat map depicting expression intensity of 50 paradoxical genes across various tumors; **(D)** SNV of paradoxical genes.

In recent years, our understanding of the molecular underpinnings of cancer has been revolutionized by the integration of genomic databases such as TCGA into cancer research (Tomczak et al., 2015). TCGA provides an extensive compilation of genetic mutations, gene expression data, and epigenetic alterations across thousands of tumors, spanning over 30 human tumor types (Weinstein et al., 2013; Tomczak et al., 2015). Through our analysis of the TCGA database, our team has determined that the role of paradoxical genes cannot be overlooked. We conducted a detailed analysis of the distribution of paradoxical genes across various tumor types, ranking them by the number of highly expressed genes within each tumor category (Figure 1A). We observed that this type of gene is ubiquitously present across various cancers. Notably, BRCA, bladder urothelial carcinoma, and kidney renal clear cell carcinoma (KIRC) exhibit the highest expression levels of these tumor suppressor genes. In contrast, kidney chromophobe (KICH), esophageal carcinoma (ESCA), and prostate adenocarcinoma (PRAD) have significantly lower expression of these genes.

This disparity suggests a complex regulatory mechanism involved in the expression of paradoxical genes, which the tumor microenvironment (TME) and specific oncogenic pathways could influence. The high expression levels of paradoxical genes in BRCA, BLCA, and KIRC suggest that these cancers possibly utilize these genes to balance between tumor suppression and oncogenic activity, potentially as a response to oncogenic stress or other cellular pressures. In contrast, the reduced expression of paradoxical genes in KICH, ESCA, and PRAD might indicate a loss of this balancing mechanism, possibly contributing to more aggressive tumor behavior. These findings provide a crucial direction for future research into the mechanisms regulating paradoxical genes and their role in cancer progression.

### 2.2 Kyoto encyclopedia of genes and genomes analysis of paradoxical genes: insights into their relationship with tumor metabolism

To investigate the functional mechanisms of paradoxical genes further, we initially screened 254 paradoxical genes for a KEGG analysis (Figure 1B). A common characteristic among these genes is their high expression in at least three tumor cell groups, correlating with improved patient prognosis. Our KEGG analysis revealed that these genes are extensively involved in various metabolism-related pathways, suggesting it as a primary mechanism through which paradoxical genes influence tumor prognosis.

Notably, liver X receptor (LXR) genes, including LXRα and LXRβ, are a few examples of this phenomenon (Han et al., 2023; Wang et al., 2023). LXRs are nuclear receptors involved in lipid metabolism, inflammation, and cholesterol homeostasis (Zelcer and Tontonoz, 2006). In the context of cancer, LXRs have demonstrated a dual role in tumor prognosis, influenced by their regulatory impact on metabolic pathways and immune responses in the tumor microenvironment (Han et al., 2023; Wang et al., 2023). LXR genes can play a role in inhibiting tumor progression (Zhang et al., 2014; Nguyen-Vu et al., 2013). This is primarily mediated through their anti-inflammatory effects within the tumor microenvironment. High expression of LXR genes causes upregulation of cholesterol efflux transporters such as ATP-binding cassette transporter A1 (ABCA1) and ABCG1, which facilitate cholesterol efflux and reduce lipid accumulation within macrophages, thus attenuating the inflammatory response associated with tumor progression (Joseph et al., 2003; Wang et al., 2006). Furthermore, LXR activation has been linked to the suppression of inflammatory cytokine production by immune cells, leading to a less conducive environment for tumor growth (Joseph et al., 2003; Fessler, 2016; Chawla et al., 2001). A study demonstrated that LXRs activation disrupts BRCA cell proliferation by downregulating the expression of genes involved in cell growth and proliferation, particularly those regulated by the E2F family of transcription factors (Nguyen-Vu et al., 2013). The activation of LXRs leads to the downregulation of key genes involved in the cell cycle, DNA replication, and other critical processes for cancer cell division (Nguyen-Vu et al., 2013). This effect is partly mediated through the regulation of E2F2, highlighting a potential mechanism by which LXRs inhibit proliferation in cancer cells (Nguyen-Vu et al., 2013).

Conversely, LXRs can promote tumor growth through several mechanisms. Their activation leads to upregulation of genes involved in lipid biosynthesis, such as SREBP-1c (Sterol Regulatory Element-Binding Protein 1c) (Okazaki et al., 2010; Jeong et al., 2021). SREBP-1c is a crucial transcription factor that enhances the expression of genes required for fatty acid and triglyceride synthesis (Okazaki et al., 2010; Jeong et al., 2021). In many cancers, particularly those with high lipid requirements like breast cancer, this can contribute to tumor cell proliferation and survival by ensuring a steady supply of essential lipids that are critical for membrane synthesis, energy storage, and signaling (Santos and Schulze, 2012; Menendez and Lupu, 2007; Swinnen et al., 2006; Zadra et al., 2013; Fukuchi et al., 2004; Vedin et al., 2009). Our research (Figure 1A) demonstrated that paradoxical genes are prevalently expressed in breast cancer, which also supports the notion that the modulation of these genes, particularly their regulation of tumor lipid metabolism, is fundamental to their anticancer effects.

In our previous investigation of clear cell renal cell carcinoma (ccRCC), we further examined the dual role of LXR (Wu et al., 2019). Our findings suggest that LXR functions as a balance gene, where both heightened and diminished expression levels can exert inhibitory effects on ccRCC progression (Wu et al., 2019). Specifically, both LXR agonists and inverse agonists inhibits cell proliferation and colony formation. The LXR agonist LXR623 downregulates low-density lipoprotein receptor (LDLR) and upregulated ABCA1, causing a decline in intracellular cholesterol and induced apoptosis (Wu et al., 2019). Conversely, the LXR inverse agonist SR9243 downregulates key FA synthesis proteins, including sterol regulatory SREBP-1c, FA synthase, and stearoyl-CoA desaturase 1(SCD1), leading to decreased FA content and apoptosis in ccRCC(Wu et al., 2019). This phenomenon illustrates that alterations in cancer metabolism are a pivotal factor in mediating the regulatory effects of paradoxical genes on tumor prognosis.

### 2.3 Expression intensity and single nucleotide variations (SNV) analysis of paradoxical genes across different tumors

Subsequently, we screened out 50 paradoxical genes for pan-cancer analysis (Figure 1C). Their common feature is that they are highly expressed in ≥4 groups of tumor cells, and are associated with better patient prognosis. The expression intensity of these genes was analyzed in 20 different tumor tissues, and we found widespread overexpression, including, but not limited to, KIRC, cholangiocarcinoma (CHOL), stomach adenocarcinoma (STAD), BLCA, PRAD, ESCA, and liver hepatocellular carcinoma (LIHC), highlighting their potential as pan-cancer prognostic markers. Interestingly, subsequent analysis of SNVs in these genes showed that mutations are infrequent, a characteristic not generally observed in traditional oncogenes (Figure 1D). This observation further supports the hypothesis that the prognostic effect of paradoxical genes is mediated through mechanisms distinct from those employed by oncogenes, potentially driving tumor evolution towards less aggressive and more treatable forms. Nonetheless, there are currently very few reports on the occurrence of highly expressed tumor suppressor genes in tumors. Uncovering previously underappreciated complexities in the relationship between gene expression and cancer prognosis is critical.

## 3 The relationship between gene abundance and protein abundance: is there always a direct correlation?

**FIGURE 2:**
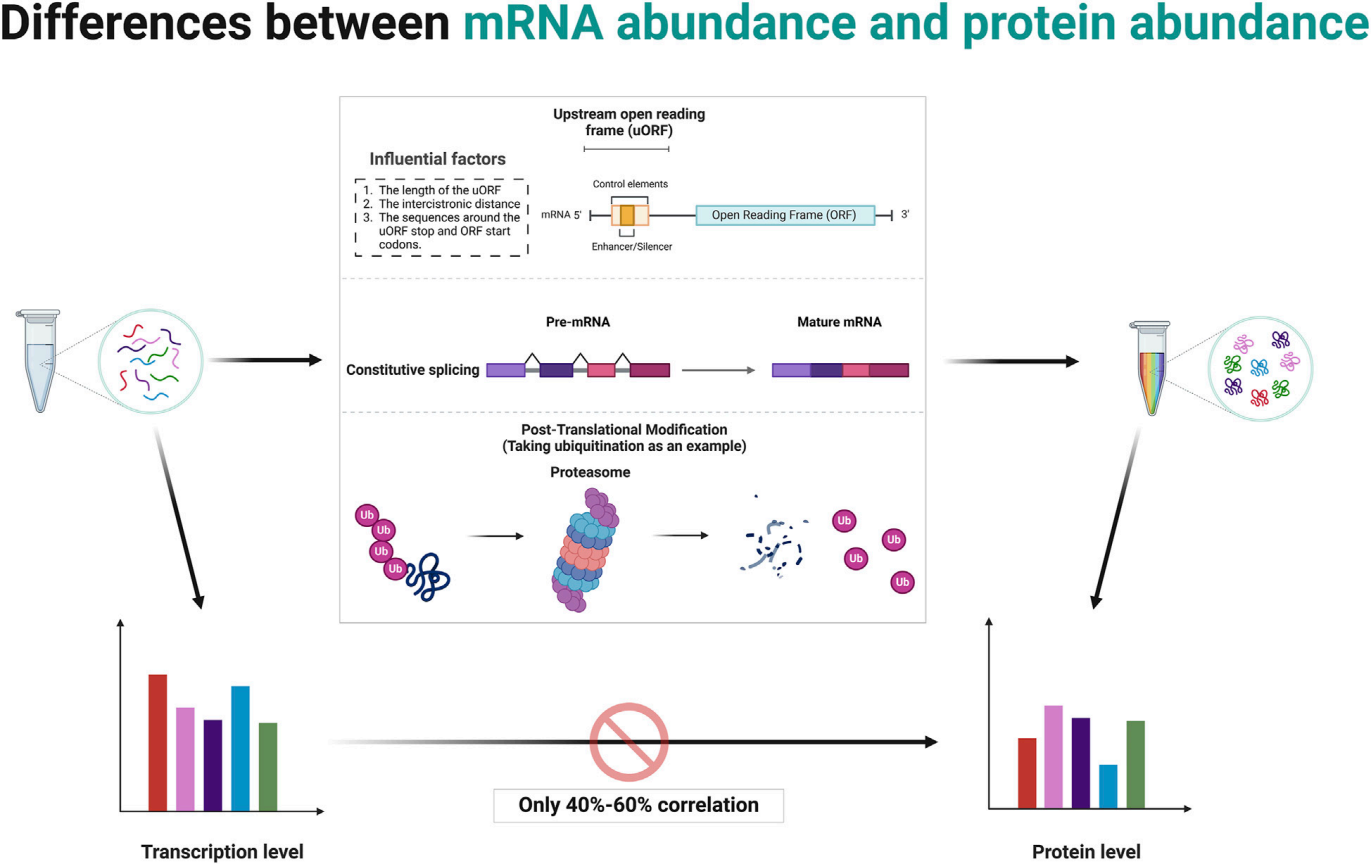
Post-transcriptional and post-translational modifications contribute to discrepancies between mRNA abundance and protein abundance. Created in BioRender. ZHAO, X. (2025) https://BioRender.com/q14t025.

The central dogma of molecular biology, formulated by Francis Crick, describes the flow of genetic information from DNA to RNA to protein through the processes of transcription and translation (Crick, 1970). While it is generally hypothesized that higher mRNA levels correlate with higher protein levels, this relationship is influenced by several factors (Vogel and Marcotte, 2012; Schwanhäusser et al., 2011; Tian et al., 2004). Post-transcriptional regulation can modify mRNA stability and translation efficiency, as well as the sequence features of the mRNA itself, such as upstream ORFs, can affect how efficiently it is translated (Barbosa et al., 2013; Sonenberg and Hinnebusch, 2009). Protein stability and degradation processes further modulate the levels of functional protein in the cell (Ciechanover and Kwon, 2015; Sherman and Goldberg, 2001). Although studies have typically shown a positive correlation between mRNA and protein levels, the variability suggests that multiple mechanisms, including translation efficiency and protein stability, are significant in determining final protein levels in biological systems (Schwanhäusser et al., 2011; Liu et al., 2016). Several studies have reported average correlation coefficients around 40%–60%, indicating that mRNA levels indicate protein abundance but are far from perfectly predictive due to various biological and methodological confounders (Vogel and Marcotte, 2012; Perl et al., 2017; Kosti et al., 2016) (Figure 2). This makes studies involving the gene level resulting from bioinformatics analysis somewhat one-sided.

### 3.1 The insights from the effect of upstream open reading frames

The concept of mRNA translation primarily involves coding mRNA into proteins by ribosomes, a process central to gene expression (Kozak, 1999; Jackson et al., 2010). Typically, this decoding focuses on the ORF that starts with a start codon (usually AUG) and ends with a stop codon (Jackson et al., 2010; Kozak, 2001). The discovery and study of uORFs have expanded our understanding of translational regulation and its complexities (Barbosa et al., 2013; Calvo et al., 2009; Wethmar, 2014). uORFs are alternative ORFs located upstream of the in the 5′untranslated region (5′UTR) of main coding sequence of an mRNA (Calvo et al., 2009). These uORFs can play a significant role in the regulation of translation of the main ORF (Barbosa et al., 2013; McGillivray et al., 2018).

uORFs are initiated when a ribosome recognizes and binds to a start codon (usually AUG, but sometimes a near-cognate codon) at the 5′UTR of an mRNA (Silva et al., 2019). The presence of a uORF upstream of the main coding sequence can alter ribosomal scanning and initiation dynamics, consequently affecting the translation of the downstream ORF (Hinnebusch et al., 2016; Morris and Geballe, 2000). After a uORF is translated, ribosomes can either dissociate from the mRNA or resume scanning for another start codon (Kozak, 2005). Several factors affect the ability of ribosomes to re-initiate translation at the downstream main ORF depends on, including the length of the uORF, the distance between the cistrons (the gap between the uORF and the main ORF), and the sequence context around the stop codon of the uORF and the start codon of the main ORF (Jackson et al., 2010; Ivanov et al., 2010; Kozak, 1987) (Figure 2). Often, efficient re-initiation is contingent upon the ribosomal retention of initiation factors during the uORF translation. The impact of a uORF on the main ORF translation can vary dramatically depending on its sequence and context (Pavitt, 2005). Some uORFs exhibit features that stall ribosomal function or slow translation, potentially enhancing or inhibiting the translation of the main ORF (Caliskan et al., 2015). For instance, Phan et al. elucidate how conserved uORFs in the 5′UTR of Polo-like kinase 4 (PLK4) mRNA play a crucial role in controlling the translation of PLK4, thereby regulating the duplication of centriole in primordial germ cells (PGCs) and preserving genomic integrity (Phan et al., 2022). This translational control mechanism prevents excessive PLK4 synthesis, vital for preventing centriole amplification and associated mitotic errors, highlighting a specific requirement for uORF in regulating the balance of PLK4 levels during germ cell development (Phan et al., 2022). A recent study by Cieśla et al. reveals that the regulation of SF3B1 protein levels through ALKBH5-driven N6-methyladenosine demethylation in the 5′UTR influences its translation, driving splicing mechanisms that impact DNA repair and epigenetic regulation (Cieśla et al., 2023). These studies demonstrate the critical role of post-transcriptional modifications in the expression of final protein.

### 3.2 The insights from alternative splicing

Alternative splicing is a post-transcriptional regulatory mechanism that contributes significantly to proteomic diversity and gene expression regulation in eukaryotic organisms (Nilsen and Graveley, 2010; Wang et al., 2008; Black, 2003; Barash et al., 2010). It involves the selective inclusion or exclusion of pre-mRNA segments (exons) during the RNA splicing process, resulting in multiple distinct mRNA transcripts from a single gene (Chen and Manley, 2009; Kornblihtt et al., 2013) (Figure 2). This process can affect the quantity as well as the functionality of the encoded proteins (Kornblihtt et al., 2013). When determining RNA abundance using technologies like RNA-seq, reads mapping to a gene are typically aggregated to estimate the overall abundance of the gene (Wang et al., 2009; Mortazavi et al., 2008). This standard approach does not differentiate between the various transcripts produced by alternative splicing (Ozsolak and Milos, 2011; Trapnell et al., 2009). Consequently, even if the total mRNA of a gene remains constant, changes in the splicing patterns can lead to proteins with significantly altered types and functions (Black, 2003; Kalsotra and Cooper, 2011). This is a critical factor to consider in gene expression analysis because while the quantitative measure (total RNA transcripts) might not show variation, the qualitative changes (different splice variants) can have profound biological ramifications (Nilsen and Graveley, 2010; Barash et al., 2010). Meanwhile, specific conditions or stimuli might induce changes in splicing patterns without altering the overall mRNA levels (Wang et al., 2008; David and Manley, 2010). Such differential splicing events can produce protein variants with differing, sometimes opposing, functions (Wang et al., 2008; David and Manley, 2010). Some splice variants may include or exclude sequences with regulatory elements affecting translation efficiency, such as internal ribosome entry sites or uORFs (Barbosa et al., 2013; Sonenberg and Hinnebusch, 2009).

In a recent study, researchers demonstrated the significant role of alternative splicing in the regulation of chromatin dynamics, particularly through the manipulation of histone deacetylase (HDAC)7 splicing downstream of T cell signaling pathways (Agosto et al., 2023). Notably, the longer HDAC7 isoform, induced by the RNA-binding protein CUGBP Elav-like family member 2, enhances the expression of key T cell surface proteins such as CD3, CD28, and CD69, highlighting the broad implications of alternative splicing on histone modification and gene regulatory mechanisms in T cells (Agosto et al., 2023). Particularly in studies related to diseases such as cancer, where splicing patterns can be drastically altered, researchers must consider the total expression level of a gene as well as the expression levels of individual splice variants.

### 3.3 The insights from post-translational regulation

Differences in protein abundance and gene abundance are largely caused by post-translational modifications (PTMs). PTMs like ubiquitination and phosphorylation can target proteins for degradation, leading to lower protein levels despite high mRNA expression (Hershko and Ciechanover, 1998; Hunter, 2007; Cohen, 2000; Ciechanover, 2005; Deshaies and Ferrell, 2001). Conversely, protein levels rise when modifications protect proteins from degradation. PTMs also modulate protein activity, producing active or inactive forms that do not directly correlate with mRNA levels (Figure 2). For instance, phosphorylated proteins often exhibit functions or stabilities different from their non-phosphorylated counterparts (Hunter, 2007; Olsen et al., 2006; Manning et al., 2002). Modifications such as phosphorylation, methylation, and acetylation also impact protein-protein interactions, altering binding affinities and affecting signaling pathways and cellular processes independent of gene expression (Ross et al., 2023; Nishi et al., 2014; Duan and Walther, 2015). The regulatory mechanism of post-translational modifications is comprehensively summarized in Table 1.

**TABLE 1:** Overview of post-translational modifications in protein regulation.

PTM	Mechanism	Regulation direction	Biological effects	Clinical significance	References
Phosphorylation	Addition of phosphate groups to amino acids (Ser, Thr, Tyr)	Activate or inhibit	Regulates enzyme activity, signal transduction, cell cycle, apoptosis	Targeting phosphorylation pathways is a strategy in cancer therapy	Hunter (2007), Singh et al. (2017)
Ubiquitination	Attachment of ubiquitin to lysine residues	Usually leads to degradation	Controls protein turnover, modulates signaling pathways, cellular stress responses	Central in neurodegenerative diseases like Alzheimer’s and Parkinson’s, and cancer therapies	Popovic et al. (2014)
Acetylation	Addition of acetyl groups to lysine residues	Can activate or stabilize proteins	Influences gene expression, enzyme activity, protein stability, and metabolic regulation	Targeted by HDAC inhibitors in cancer treatment	Shvedunova and Akhtar (2022), Drazic et al. (2016), He et al. (2023)
Methylation	Addition of methyl groups to lysine or arginine	Can activate or repress	Affects protein interaction, stability, DNA binding, and transcriptional regulation	Targeted by drugs that modify methylation dynamics (inhibitors of methyltransferases and demethylases)	Greer and Shi (2012), Dawson and Kouzarides (2012), Klose and Zhang (2007)
Sumoylation	Addition of SUMO proteins to lysine residues	Typically inhibits	Regulates nuclear-cytosolic transport, transcriptional activity, DNA repair	Implicated in cancer and heart disease	Flotho and Melchior (2013), Geiss-Friedlander and Melchior (2007), Chang and Yeh (2020)
Prenylation	Attachment of lipid groups (farnesyl or geranylgeranyl) to cysteine residues at the C-terminus of proteins	Generally activates	Facilitates membrane attachment, affects protein localization and function in signaling	Targeted in anti-cancer therapies, especially in Ras-related cancers	Jung and Bachmann (2023), Baranyi et al. (2020)

PTM, post-translational modifications; HDAC, histone deacetylase; SUMO, small ubiquitin-like modifier; Ser, serine; Thr, threonine; Tyr, tyrosine.

Integrative multi-omics analysis highlights the significant impact of PTMs on the differences in protein and gene levels, particularly during the human cell cycle (Parkes and Niranjan, 2019). While mRNA and translation data explain some variations in protein abundance, the remaining inconsistencies are primarily due to PTMs, which adjust protein levels post-synthesis (Parkes and Niranjan, 2019). In a study focusing on triple-negative breast cancer (TNBC), researchers identified tumor endothelial marker 8 (TEM8) as a key indicator of breast tumor-initiating cells (Xu et al., 2021). The study also highlighted the binding of estrogen receptor α to the promoter region of the ubiquitin E3 enzyme ankyrin repeat and SOCS box containing 10 (ASB10). It also activates ASB10 transcription, and ASB10 interacts with TEM8, thereby affecting the ubiquitination of TEM8 and ultimately affecting the TEM8 protein level (Xu et al., 2021). Thus, Indirect evidence indicates that variations in TEM8 mRNA and protein expression across BRCA subtypes may be attributed to post-translational modifications (Xu et al., 2021).

## 4 Regulation of tumor immune microenvironment by paradoxical genes

**FIGURE 3:**
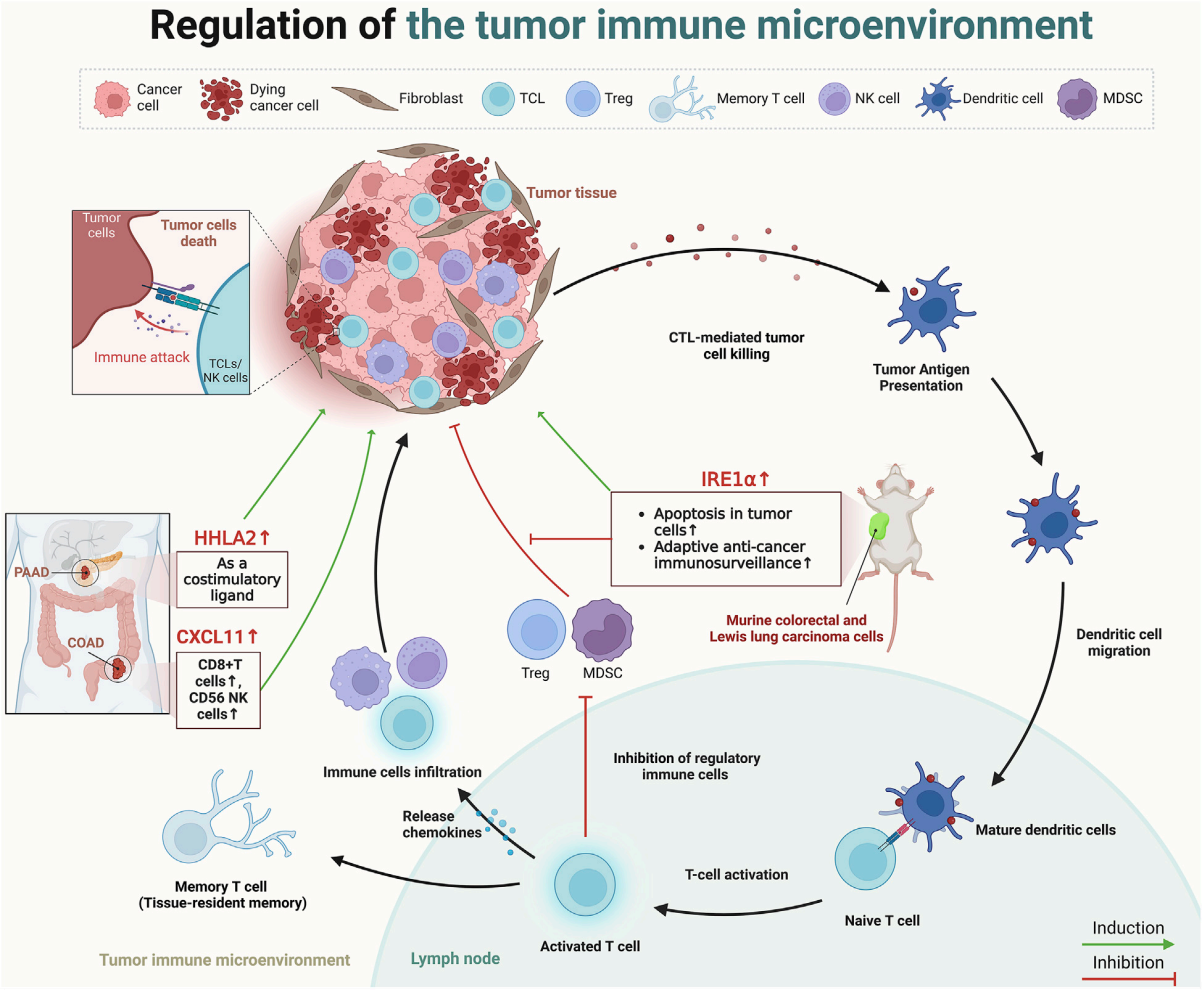
The role of paradoxical genes in regulating the TIME. Created in BioRender. ZHAO, X. (2025) https://BioRender.com/k01e299.

The process of TIME begins with the recognition of tumor-specific antigens by antigen-presenting cells, like dendritic cells, which capture and present these neoantigens to naïve T cells in lymph nodes, thereby initiating T-cell activation (Mellman et al., 2011). This activation triggers a series of immune responses, including the release of chemokines that attract more effector immune cells (such as CTLs, NK cells, and macrophages) to the tumor site, effectively infiltrating the tumor (Joyce and Fearon, 2015). Within the TIME, a pivotal change occurs as effector T cells reprogram immunosuppressive cells and alter the metabolic environment, diminishing the suppressive function of regulatory immune cells such as regulatory T cells (Tregs), myeloid-derived suppressor cells (Ho et al., 2015). This culminates in the direct cytotoxic attack on tumor cells by CTLs and NK cells, utilizing mechanisms like perforin and granzyme release to induce tumor cell apoptosis (Trapani and Smyth, 2002). Furthermore, some activated T cells differentiate into memory T cells, providing long-term surveillance and a rapid response mechanism against tumor recurrence (Sallusto et al., 2004) (Figure 3).

Recently, several scholars have dedicated their research efforts to understanding how paradoxical genes influence prognosis through the regulation of the TIME. Cao et al. analyzed data from TCGA and the gene expression omnibus, revealing that the expression of C-X-C motif chemokine ligand (CXCL)11 was elevated in colon cancer tissues compared to healthy tissues, and higher levels of CXCL11 correlated with improved survival outcomes (Cao et al., 2021). Furthermore, assessment of three independent datasets, including TCGA and two single-cell RNA sequencing datasets from Gene Expression Omnibus, in addition to immunohistochemistry data from a COAD patient cohort demonstrated that this tumor suppressor effect possibly due to its association with an increased presence of antitumor immune cells (CD8^+^ T cells, and CD56 NK cells), underscored CXCL11’s role in modulating the TIME (4). Furthermore, HERV-H LTR-associating 2 (HHLA2), a newly identified member of the B7 immune checkpoint family, is also a typical paradoxical gene (Yan et al., 2019). HHLA2 is minimally expressed in normal pancreatic tissues but shows significant upregulation from precancerous stages to invasive pancreatic ductal adenocarcinoma (PDAC), according to immunohistochemistry analyses on tissue microarrays (Yan et al., 2019). In 77.17% of PDAC cases, the expression of HHLA2 is strongly associated with an improved post-surgical prognosis, indicating functions of HHLA2 as a costimulatory ligand in pancreatic cancer, activating CD8^+^ T cell proliferation and improving patient prognosis (Yan et al., 2019). A subsequent study also presented a similar point; immunohistochemical analysis on tissue micro-arrays from surgically resected tumors of 122 pancreatic and 72 ampullary cancer patients revealed HHLA2 expression in 67% of pancreatic and 93% of ampullary tumors, associating enhanced expression with improved post-surgical outcomes, including delayed cancer recurrence and improved cancer-specific survival (Boor et al., 2020). Similarly, the study by Martinez-Turtos et al. highlights that overexpression of inositol-requiring enzyme 1α (IRE1α) in murine colorectal and Lewis lung carcinoma cells in syngeneic immunocompetent mice, leads to a tumor-suppressive phenotype (Martinez-Turtos et al., 2022). This anti-tumoral effect is attributed to the RNAse activity of IRE1α, which induces apoptosis in tumor cells, enhances adaptive anti-cancer immunosurveillance through XBP1 mRNA splicing, and regulates IRE1-dependent degradation of RNA (RIDD) (Martinez-Turtos et al., 2022). However, in addition to the tumor suppressive role of IRE1α, its tumor-promoting role is also evident in preclinical models of various cancers, including TNBC, PDAC, and colon cancer (Harnoss et al., 2020; Garcia-Carbonero et al., 2018; Xie et al., 2019). This duality suggests that the impact of Paradoxical genes on cancer prognosis may be multifaceted and not solely affected by the TIME (Figure 3).

## 5 Dual roles of paradoxical genes and associated signaling pathways in tumors

**FIGURE 4:**
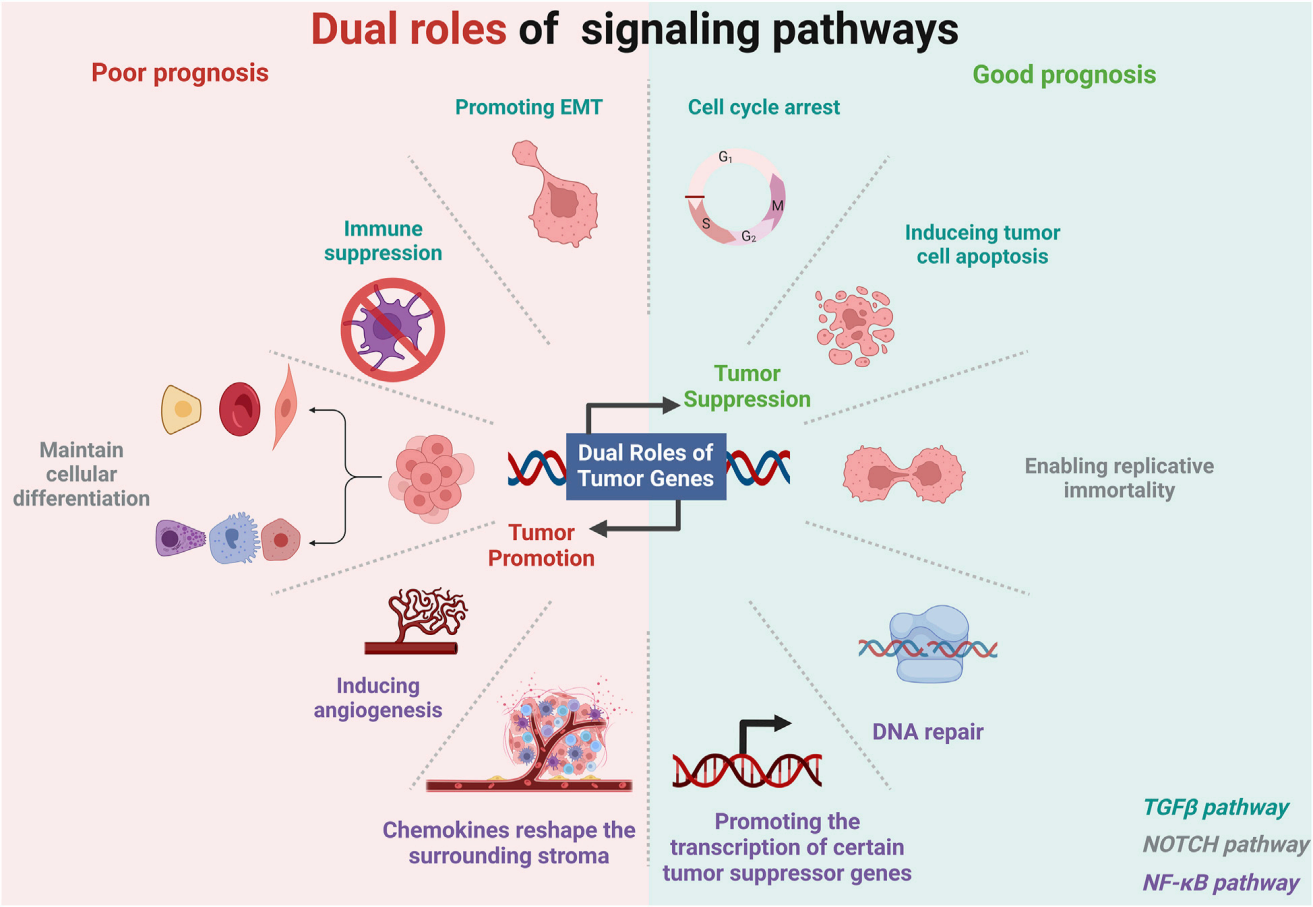
Dual roles of gene pathways in different tumor environments. Created in BioRender. ZHAO, X. (2025) https://BioRender.com/f74o594.

Certain genes and their associated signaling pathways exhibit bidirectional effects on tumor prognosis, transitioning between inhibiting and promoting tumor progression. This dualistic behavior is also one of the key factor contributing to the emergence of paradoxical genes (Hanahan and Weinberg, 2011; Sadikovic et al., 2008). This biphasic regulatory effect can depend on various factors, including tumor stage, tumor-specific expression, and TME (Hanahan and Weinberg, 2011; Plaks et al., 2015; Gerlinger et al., 2012; Marusyk et al., 2012; Bray, 2016). The impact of gene expression can vary by cancer type and the tissue of origin (Stratton et al., 2009). Genes beneficial in one type of cancer might be deleterious in another (Vogelstein and Kinzler, 2004; Hanahan and Weinberg, 2011; Sadikovic et al., 2008). The role of E-cadherin in tumor suppression is well-established in BRCA due to its function in maintaining cell-cell adhesion and inhibiting metastasis (Padmanaban et al., 2019). However, in other cases, such as gastric cancer, the expression of can be associated with different outcomes based on additional factors like the presence of specific mutations or the overall state of cellular adhesion molecules (Becker et al., 1994). We focuses on signaling pathways with biphasic regulatory effects, including the TGFβ, NOTCH, and NF-κB pathways (Figure 4). Genes associated with these pathways are key contributors to the development of paradoxical genes, which exhibit peculiar behaviors during gene expression and regulation.

## 6 Stage-specific expression of paradoxical genes: insights from the transforming growth factor-β signaling pathways

The expression of tumor suppressor genes exhibits significant variability across different cancer stages, contributing to the phenomenon of paradoxical genes (Sherr and McCormick, 2002). For instance, in the early stages of cancer, key genes like TP53 and RB1 play a crucial role in maintaining cellular integrity by regulating DNA damage repair and controlling cell cycle progression (Sherr and McCormick, 2002). However, as the tumor evolves, these genes often become inactivated due to mutations, deletions, or epigenetic modifications, leading to unchecked cell proliferation and advancement to more aggressive stages (Li et al., 1997; Cantley and Neel, 1999). PTEN, which regulates the PI3K/AKT signaling pathway, is commonly altered in cancers such as prostate and breast cancer, thereby facilitating tumor growth and survival (Li et al., 1997; Cantley and Neel, 1999; Salmena et al., 2008). In later stages, the suppression of tumor suppressor genes can precipitate metastasis and resistance to treatment, severely worsening the prognosis (Valastyan and Weinberg, 2011; Gottesman, 2002; Hanahan and Weinberg, 2000). Notably, the TGF-β pathway is recognized for its dual role in oncogenesis, acting as a tumor suppressor in initial stages while potentially fostering cancer progression in advanced stages (Massagué, 2008). Within this pathway, SMAD family members, including SMAD2 and SMAD4, are pivotal in relaying TGF-β signals that suppress cell division (Derynck and Zhang, 2003).

The TGFβ pathway suppresses tumors in the early stages of tumor development mainly by maintaining cellular homeostasis (including cell cycle arrest and apoptosis) and preventing uncontrolled cell proliferation (Colak and Ten Dijke, 2017). TGFβ regulates the cell cycle by inhibiting the transition from the G1 phase to the S phase, thereby preventing DNA replication and cell division, which is facilitated through the upregulation of cyclin-dependent kinase (CDK) inhibitors, which deactivate CDKs essential for cell cycle progression (Derynck, 1994; Decker et al., 2021). TGFβ also induces apoptosis, or programmed cell death through the activation of death-associated proteins and modulation of apoptosis-related genes, eliminating cells with potentially harmful mutations (Zhao et al., 2018; Schulte-Hermann et al., 1992). It also maintains cellular differentiation and proper morphology, thereby inhibiting the epithelial-mesenchymal transition (EMT), a critical process in cancer metastasis (Hao et al., 2019). Furthermore, TGFβ exerts anti-inflammatory effects within the cellular environment, regulating immune cell activity and cytokine production to suppress chronic inflammation, thus preventing tumor growth (Viel et al., 2016; Coussens and Werb, 2002).

In advanced cancer, TGF-β primarily acts as a tumor promoter. TGFβ promotes the EMT, a critical process for metastasis, by regulating transcription factors like Snail, Slug, and Twist that modify adhesion and migration properties of the cell (Peng et al., 2022; Wang et al., 2013; Ang et al., 2023). Simultaneously, TGF-β exerts systemic immune suppression and inhibits host immunosurveillance and also regulates the infiltration of inflammatory/immune cells and cancer-associated fibroblasts in the TME, causing direct changes in tumor cells (Yang et al., 2010). Neutralizing TGF-β enhances CD8+ T-cell- and NK-cell-mediated anti-tumor immune responses and increases the neutrophil-attracting chemokine production, leading to the recruitment and activation of neutrophils with an antitumor phenotype (Yang et al., 2010). It also interacts with cancer-associated fibroblasts and mesenchymal stem cells within the TME to remodel the extracellular matrix, increasing tumor stiffness and spreading cancer (Arima et al., 2023) (Figure 4).

The development of inhibitors that target TGFβ signaling is a promising treatment approach for cancers where TGFβ promotes tumor growth and metastasis (Derynck and Akhurst, 2007; Herbertz et al., 2015). These inhibitors generally block TGFβ receptors, preventing the downstream signaling cascades that lead to oncogenic effects (Herbertz et al., 2015). SB-431542 was initially identified as a potent and specific inhibitor of the activin receptor-like kinase (ALK)4, ALK5, and ALK7 type I receptors of the TGF-β superfamily, effectively and selectively inhibiting activin and TGF-β signaling without impacting BMP signaling or other divergent pathways like extracellular signal-regulated kinase (ERK), c-Jun N-terminal kinase (JNK), or p38 mitogen activated protein kinase (Inman et al., 2002). In recent years, galunisertib (LY2157299 monohydrate), a selective, small molecule that can be taken orally to inhibit TGF-β receptor I kinase, exhibits antitumor activity across various cancer models, including breast, colon, lung, and HCC, by specifically downregulating SMAD2 phosphorylation and inhibiting the canonical TGF-β pathway (Herbertz et al., 2015). This drug is currently being evaluated in clinical trials in an intermittent dosing regimen (14 days on/14 days off, on a 28-day cycle) as part of monotherapy or in combination with other antitumor treatments to balance efficacy and safety, targeting cancers with high unmet medical needs like glioblastoma, pancreatic cancer, and HCC (Herbertz et al., 2015; Akhurst and Hata, 2012; Nadal et al., 2023). Clinical trials investigating combinations of TGFβ inhibitors with programmed cell death protein 1(PD-1)/programmed death-ligand 1(PD-L1) inhibitors are exploring this approach, showing promising results in improving anti-tumor immunity and patient outcomes (Wrzesinski et al., 2007). Hence, understanding the tumor stage and the specific role of TGFβ is crucial to determining when and how to target this pathway effectively. Personalizing treatment based on the genetic and molecular profiles of individual tumors could optimize the efficacy of TGFβ inhibitors and minimize adverse effects.

## 7 Tumor-specific expression of paradoxical genes: insights from the NOTCH signaling pathway

The NOTCH signaling pathway is a crucial cell communication mechanism that influences various biological processes, such as differentiation, proliferation, apoptosis, and stem cell maintenance (Bray, 2016). This pathway involves NOTCH receptors (NOTCH1-4) interacting with delta-like (DLL1, DLL3, DLL4) or Jagged (JAG1, JAG2) ligands on adjacent cells, initiating proteolytic cleavages that release the NOTCH intracellular domain (NICD) (Bray, 2016). This domain moves to the nucleus, where it converts recombination signal binding protein for immunoglobulin kappa J region from a repressor to an activator, with mastermind-like proteins, initiating transcription of target genes families like HES and HEY (Bray, 2016). The finely tuned regulation of this pathway, which includes endocytic trafficking and post-translational modifications, is essential for maintaining cellular and tissue homeostasis (Fortini, 2009). The dual nature of NOTCH signalling in cancer biology—acting as a tumor suppressor in some contexts while promoting tumor progression in others—underscores the complexity of its signaling pathways and their diverse effects on cancer etiology and progression (Valdez and Xin, 2013) (Figure 4).

In specific cancers like those of skin and liver, NOTCH signaling is essential for maintaining cellular differentiation and tissue architecture (Panelos and Massi, 2009; Kawaguchi and Kaneko, 2021). Specifically, in squamous cell carcinoma, increased NOTCH signaling is associated with reduced tumor formation and progression (Panelos et al., 2008). Impaired NOTCH signaling, as demonstrated by the expression of the pan-NOTCH inhibitor dominant negative mastermind-like (DNMAML)1 in conditional transgenic mice, leads to hyperplastic epidermis and spontaneous development of cutaneous squamous cell carcinoma (SCC) and actinic keratoses, suggesting a protective role of canonical NOTCH signaling against cutaneous SCC (Proweller et al., 2006). Meanwhile, NOTCH1 signaling significantly inhibits the growth of HCC by inducing cell cycle arrest at the G (0)/G (1) phase and promoting apoptosis, by downregulating key cell cycle proteins and upregulating p21 and p53, while also suppressing antiapoptotic B-cell lymphoma 2(Bcl-2) expression (Giovannini et al., 2016; Giovannini et al., 2014; Kim et al., 2017; Qi et al., 2003; Viatour et al., 2011). However, the role of NOTCH signaling is paradoxically reversed in other types of cancers. In T-cell acute lymphoblastic leukemia (T-ALL) and certain breast cancers, NOTCH activation enhances cell proliferation, survival, and stemness, thereby promoting tumor growth and survival (Weng et al., 2004; Reedijk et al., 2005). Besides, the NOTCH signaling pathway also presents a biological dual nature widely in various cancers (Table 2).

**TABLE 2:** Summary of the tumor-promoting or tumor-inhibiting effects of the NOTCH signaling pathway in various cancers.

Effect on tumors	Cancer type	Predominant NOTCH receptor	Role of notch signaling pathway	Signaling pathways involved	Mechanism of action	References
Promotion	Breast cancer	NOTCH1, NOTCH2, NOTCH3	Promotes cell proliferation, survival, stemness, and contributes to treatment resistance and metastasis in breast cancer	JAG1-NOTCH1, NOTCH1-PTEN-ERK1/2, HER2, PKCα-JAG1-NOTCH, Notch3, FYN-STAT5-NOTCH2	Enhances proliferation, stem cell survival, and metastasis; modulates resistance to treatments; promotes aggressive tumor traits	Reedijk et al. (2005), Baker et al. (2018), Pandya et al. (2016), Saran et al. (2023), Leontovich et al. (2018), Lee et al. (2018)
	Colon cancer	NOTCH1, NOTCH3	Activates pathways that facilitate tumor cell survival and maintain stem cells	Wnt/β-catenin, Hedgehog	Enhances tumor cell survival, supports stem cell maintenance, and represses secretory cell differentiation	Ishiguro et al. (2017), Bertrand et al. (2012), Brzozowa-Zasada (2022)
	T lymphoblastic neoplasms	NOTCH1	Activates oncogenic pathways in T-cell neoplasms, contributes to chromosomal translocations, and influences transcriptional regulation in T-cell leukemia	Chromosomal translocations involving TAN-1 (NOTCH1), convergence with transcription factors like Ikaros	Induces oncogenic transformations in T-cell neoplasms, facilitates leukemia progression, and influences transcriptional regulation	Ellisen et al. (1991), Bellavia et al. (2007)
	T-ALL	NOTCH1, NOTCH3	Serves as a biomarker and strengthens NOTCH signaling in T-ALL, indicating aggressive disease and supporting leukemia maintenance	Related to NOTCH signaling pathways, specifically NOTCH3/Jagged1 signaling	Serves as a biomarker indicating aggressive disease, reinforces Notch signaling, and promotes disease progression and maintenance	Bardelli et al. (2021), Pelullo et al. (2014)
	NSCLC	NOTCH1, NOTCH3	Activates EGFR and other pathways, contributes to metastasis and stemness, and is associated with poor prognosis in NSCLC	EGFR, RFC4/Notch1 signaling, Notch3 overexpression	Enhances NSCLC metastasis, stemness, and tumor progression; correlates with poor prognosis	Pancewicz-Wojtkiewicz (2016), Liu et al. (2021b), Ye et al. (2013)
	CCRCC	NOTCH1, NOTCH3	Influences renal cancer cell proliferation and increases metastasis risk and tumor growth	Cell cycle regulation and HIF-2α, PI3K/Akt signaling	NOTCH3 regulates cell cycle progression and HIF-2α; NOTCH1 linked to increased metastasis risk and promotes tumor growth via PI3K/Akt	Han et al. (2020), Ai et al. (2012), Xu et al. (2012)
	Glioma	NOTCH1, NOTCH3	Regulates several pathways to promote tumor growth, stemness, and invasion in gliomas	EGFR/c-myc, TGFβ/Hippo/Notch	Enhances glioma cell invasion, self-renewal, and growth; interacts with multiple pathways to promote tumor development	Zhao et al. (2017), Xing et al. (2015), Pierfelice et al. (2011), Yi et al. (2019)
	Prostate cancer	NOTCH1, NOTCH3	Influences tumor growth and progression in prostate cancer by altering cellular behaviors and gene expression	Changes in NOTCH expression during development and tumorigenesis	Supports tumor growth and development through modulation of signaling pathways	Carvalho et al. (2014), Shou et al. (2001)
Both	HNSCC	NOTCH1	Influences tumor cell plasticity and contributes to HNSCC progression and suppression	EGFR, γ-secretase, Mutational landscape	Modulates tumor cell behavior and survival through interactions with EGFR and γ-secretase; linked to survival outcomes	Kałafut et al. (2021), Li et al. (2007), Stransky et al. (2011), Wirth et al. (2018)
Suppression	HCC	NOTCH1, NOTCH3	Inhibits HCC growth through cell cycle arrest, apoptosis induction, and modulation of key molecular pathways	Hippo, Wnt/β-catenin, JNK, Cyclin G1, MDM2, miR-221	Induces cell cycle arrest and apoptosis; suppresses tumor growth by modulating signaling pathways	Giovannini et al. (2016), Giovannini et al. (2014), Kim et al. (2017), Qi et al. (2003), Viatour et al. (2011), Sui et al. (2017)
	Cervical cancer	NOTCH1	Downregulation of Notch1 signaling is required for sustained HPV-E6/E7 expression in cervical cancer	HPV-E6/E7 expression, Notch1 signaling	Sustains HPV-E6/E7 expression and facilitates malignant transformation by specifically modulating Notch1 signaling	Talora et al. (2002)
	Neuroblastoma	NOTCH1, NOTCH2	Induces growth arrest in neuroblastoma cells by activating NOTCH signaling	Delta-Notch, Phox2B mutations	Induces cell cycle arrest and inhibits neuroblastoma cell growth through activation of Notch signaling	Zage et al. (2012), van Limpt et al. (2005)

T-ALL, T-cell acute lymphoblastic leukemia; NSCLC, non-small cell lung cancer; CCRCC, clear cell renal cell carcinoma; HCC, hepatocellular carcinoma; HNSCC, head and neck squamous cell carcinoma; TGF, transforming growth factor; HPV, human papillomavirus; EGFR, epidermal growth factor receptor; HIF, hypoxia-inducible factor; PKCα, protein kinase C α Phox2B, paired-like homeobox 2B; RFC4, replication factor C subunit 4; PI3K, phosphoinositide 3-kinase.

## 8 The impact of environmental sensitivity on the formation of paradoxical genes

The environmental sensitivity of suppressor genes refers to the fact that the expression and function of these genes are affected by the TME, including factors such as hypoxia and acidity, which in turn affects cancer progression and cellular behavior (Gatenby and Gillies, 2004; Webb et al., 2011). Additionally, immune cells within this microenvironment release a variety of cytokines and growth factors that significantly impact cancer dynamics (Coussens and Werb, 2002; Grivennikov et al., 2010; de Visser et al., 2006). Specifically, certain immune-derived factors may suppress tumor cells, while others might activate signaling pathways that induce tumor cells (Coussens and Werb, 2002; Grivennikov et al., 2010; de Visser et al., 2006). These regulatory effects of environmental sensitivity on tumor suppressor genes undoubtedly contributed to the emergence of paradoxical genes.

### 8.1 Insights from hypoxia

Hypoxia within the TME critically influences the progression of cancer by modulating tumor suppressor genes, primarily via the stabilization of hypoxia-inducible factors (HIFs) such as HIF-1α and HIF-2α (Semenza, 2003; Keith et al., 2011). Under low oxygen conditions, these transcription factors translocate to the nucleus, activating genes that drive angiogenesis, metabolism, cell survival, and invasion (Semenza, 2003). The suppression of the VHL gene under hypoxic conditions leads to unregulated HIF activity, promoting the secretion of angiogenic factors like vascular endothelial growth factor and platelet-derived growth factor, which are instrumental in tumor growth and proliferation (Semenza, 2003). Hypoxia plays a significant role in the regulation of tumor suppressor genes. For instance, Chen et al. discovered that Hypoxia-inducible HIF-1α directly interacts with Mdm2, enhancing the *in vivo* association between p53 and HIF-1 alpha and acting as a mediator in their indirect interaction, which is crucial for the stabilization and activation of p53 in response to hypoxic stress (Chen et al., 2003). Furthermore, they found that HIF-1 alpha inhibits the Mdm2-mediated ubiquitination and nuclear export of p53, thereby protecting p53 from degradation and facilitating its role in transcriptional activation under hypoxic conditions (Chen et al., 2003). Additionally, PTEN, a crucial regulator of the PI3K/AKT signaling pathway, is downregulated by microRNAs like miR-21, which are themselves upregulated under hypoxic conditions. This indicates that hypoxia indirectly plays a significant role in the regulation of PTEN through the modulation of microRNA levels (Cascio et al., 2010; Kulshreshtha et al., 2007; Meng et al., 2007).

Hypoxic stress within solid tumors profoundly influences epigenetic regulation, particularly affecting DNA methylation and histone modifications (Shahrzad et al., 2007; Krieg et al., 2010). For instance, Watson et al. investigated the effects of chronic hypoxia in prostate cells and identified significant epigenetic changes, including increased global DNA methylation and H3K9 histone acetylation, associated with an altered cellular phenotype characterized by enhanced apoptotic resistance, cellular senescence, and increased invasiveness (Watson et al., 2009). These findings suggest that chronic hypoxia induces genome-wide adjustments in DNA methylation and histone modifications, potentially promoting and maintaining a hypoxic-adapted cellular phenotype that may contribute to tumor development (Watson et al., 2009). Meanwhile, Krieg et al. demonstrated that HIF-1α regulates the histone demethylase JMJD1A, which enhances the expression of hypoxia-responsive genes and promotes tumor growth (Krieg et al., 2010). This study highlights the critical role of HIF-1α in modifying histone enzymes under hypoxic conditions, thereby contributing to the dynamic regulation of gene expression in cancer cells (Krieg et al., 2010). These epigenetic modifications could further facilitate the emergence of paradoxical genes by modulating the expression diversity of tumor suppressor genes.

These molecular alterations under hypoxic stress lead to severe consequences, including enhanced angiogenesis, facilitating metastatic spread, altered cellular metabolism favoring cancer cell survival in low oxygen conditions, and increased resistance to conventional therapies (Semenza, 2003; Harris, 2002; Semenza, 2010). Targeting these adaptations has led to novel therapeutic strategies aimed at the hypoxic niche—such as the development of inhibitors that block HIFs, strategies to restore the functions of inactivated tumor suppressor pathways, and the use of hypoxia-activated prodrugs (Semenza, 2003; Wilson and Hay, 2011; Brown and Wilson, 2004; Wigerup et al., 2016). Additionally, addressing the downstream effects of tumor suppressor gene suppression, such as the use of PI3K inhibitors in cases of PTEN loss, offers a refined approach to disrupt the survival mechanisms employed by tumor cells in the hypoxic TME (Sansal and Sellers, 2004; Janku et al., 2018). These approaches leverage the modulation of tumor suppressor genes within the hypoxic tumor microenvironment to enhance the efficacy of cancer treatment.

### 8.2 Insights from acidosis

Acidosis within the TME significantly also impacts the cancer cell behavior (Webb et al., 2011; Damaghi et al., 2013). This condition stems from metabolic alterations in tumor cells, notably the high rates of glycolysis leading to excessive lactic acid production, even in the presence of oxygen (Webb et al., 2011; Gillies et al., 2008). This metabolic shift results in an accumulation of lactic acid, leading to a marked decrease in pH within the surrounding tissue (Gillies and Gatenby, 2007). Meanwhile, Riemann et al. demonstrated that an acidic tumor microenvironment induces reactive oxygen species (ROS) formation which then activate mitogen-activated protein kinases (MAPK) signaling in cancer cells (Riemann et al., 2011). This activation leads to phosphorylation of the transcription factor CREB via p38, altering transcriptional activity and potentially sustaining tumorigenic changes even after cells return to a normal environment (Riemann et al., 2011). Additionally, the acidic environment can further lead to epigenetic changes, affecting DNA methylation and histone modifications, potentially leading to the silencing of tumor suppressor genes or the activation of oncogenes (Thorne et al., 2009; Kulis and Esteller, 2010). These insights highlight the regulatory influence of pH factors on tumor prognosis and related signaling pathways, thereby creating conditions that are favorable for the emergence of paradoxical genes.

### 8.3 Insights from immune signaling pathways modulation

Immune modulation can have profound effects on the expression and function of tumor suppressor genes (Oeckinghaus et al., 2011). A key player in this regulatory network is the NF-κB signaling pathway, which orchestrates responses that can either inhibit or promote tumor progression based on the surrounding cellular context (Karin et al., 2002; Baud and Karin, 2009). NF-κB promotes oncogenesis by upregulating the expression of genes that promote cell proliferation and inhibit programmed cell death. Key targets include genes encoding anti-apoptotic proteins, such as Bcl-2, B-cell lymphoma-extra large (Bcl-xL), and inhibitor of apoptosis proteins, cell cycle regulators (such as cyclin D1 and c-Myc), and growth factors that together foster an environment conducive to the survival and proliferation of cancer cells (Annunziata et al., 2007; Karin et al., 2002; Baud and Karin, 2009). This role of NF-κB has been extensively documented in cancers like multiple myeloma, where it contributes directly to the survival and proliferation of malignant cells under chemotherapeutic stress (Annunziata et al., 2007). Additionally, NF-κB is a critical regulator of the TME by stimulating the production of pro-inflammatory cytokines and chemokines such as TNF-α, interleukin (IL)-6, and IL-8. These molecules aid in reshaping the surrounding stroma, promoting angiogenesis, and facilitating tumor cell invasion and metastasis (Karin, 2006). Furthermore, NF-κB helps recruit and activate various immune cells within the TME that support tumor growth rather than combat it, thus contributing to tumor progression and the suppression of effective anti-tumor immune responses (Karin, 2006). NF-κB also contributes to the ability of tumor cells to evade immune surveillance. It modulates the expression of molecules affecting the immune response, such as major histocompatibility complex molecules and PD-L1, a ligand for the PD-1 receptor on T cells, which inhibits T cell function. NF-κB promotes an immunosuppressive microenvironment by enhancing the expression of PD-L1 on tumor cells, allowing tumor cells to escape detection and destruction by the immune system (Greten and Karin, 2004).

The NF-κB also plays key role as a suppressor of tumor development under certain contexts (Perkins, 2012). This suppression is primarily evident during the early stages of cancer and involves mechanisms that maintain cellular homeostasis and inhibit malignant transformations (Perkins, 2012). NF-κB contributes to genomic stability maintenance by regulating the expression of genes involved in DNA repair and cell cycle checkpoints. This function prevents the accumulation of genetic mutations that could otherwise lead to oncogenesis (Volcic et al., 2012). NF-κB can also induce cellular senescence, a permanent cell cycle arrest that functions as a barrier against the proliferation of potentially cancerous cells. This dual role of promoting DNA repair and senescence helps to suppress early tumor development and progression (Janssens and Tschopp, 2006). In specific cellular contexts, NF-κB can activate the transcription of certain tumor suppressor genes. For example, NF-κB induces the expression of GADD45β, a stress-response gene that plays a crucial role in DNA repair and cell cycle regulation (Jarome et al., 2015; Al Tarrass et al., 2024; De Smaele et al., 2001). By activating such genes, NF-κB contributes to the activation of mechanisms that can curb uncontrolled cell growth and promote apoptotic pathways in cells that have undergone malignant transformation (De Smaele et al., 2001) (Figure 4).

The ubiquitous role of the NF-κB signaling pathway in cancer makes it a significant target for therapeutic intervention (Karin, 2006). Current therapeutic modalities focus on NF-κB aim to mitigate its tumor-promoting actions while preserving or enhancing its tumor-suppressive capabilities (Karin and Greten, 2005). Therapeutic agents that inhibit NF-κB can potentially reduce tumor-associated inflammation and diminish the supportive TME that fosters cancer cell survival and metastasis (Moreau et al., 2011). For instance, the use of proteasome inhibitors such as bortezomib, which prevents the degradation of IκB (inhibitor of NF-κB), thus inhibiting NF-κB activation, has been found effective in treating multiple myeloma by reducing NF-κB mediated survival signals (Moreau et al., 2011). Additionally, strategies to modulate the role of NF-κB in immune suppression are being explored; modulation of NF-κB is being studied in the context of enhancing the effectiveness of immunotherapies, such as checkpoint inhibitors (Lim et al., 2016). By suppressing NF-κB-induced PD-L1 expression on tumor cells, these therapies can enhance T-cell activity against tumors, through a dual approach by directly inhibiting tumor cell survival mechanisms while boosting anti-tumor immunity (Lim et al., 2016). These examples illustrate the importance of the therapeutic targeting of NF-κB is significant due to its ability to alter the TME, reduce tumor resistance to conventional therapies, and improve the outcomes of immunotherapeutic approaches (Karin, 2006; Greten and Karin, 2004; Gilmore and Herscovitch, 2006).

## 9 Conclusion

Traditionally, tumor suppressor genes such as TP53, retinoblastoma 1, and PTEN are well-known for their roles in regulating vital cellular processes, including DNA repair, cell cycle progression, and apoptosis (Salmena et al., 2008; Vogelstein et al., 2000; Kaelin, 1997). The TP53 gene, often described as the “guardian of the genome,” is implicated in nearly half of the occurrence of all human cancers due to its critical functions in DNA repair and cell cycle regulation (Vogelstein et al., 2000). Similarly, RB1 controls the G1/S transition in the cell cycle, and PTEN counteracts the PI3K/AKT signaling pathway to influence cell survival (Salmena et al., 2008; Kaelin, 1997). Typically, the loss of function in these genes, whether through mutations, deletions, or epigenetic alterations, lead to the uncontrolled cell growth that characterizes cancer (Salmena et al., 2008; Vogelstein et al., 2000; Kaelin, 1997).

Contrary to long-standing scientific beliefs that these genes are rarely overexpressed in tumor tissues, recent advancements and the continuous enrichment of gene network databases, including TCGA have confirmed that the potential overexpression of tumor suppressor genes in such tissues. This phenomenon may be influenced by variations in tumor mutational burden and the interplay between compensatory regulatory mechanisms and the tumor microenvironment. Tumors with high tumor mutational burden often exhibit increased genomic instability, which can lead to the upregulation of tumor suppressor genes as part of a compensatory response. This has been consistently demonstrated in database studies as well as in basic experimental research. This phenomenon has been consistently demonstrated through both database analyses and fundamental experimental studies. While many studies focus primarily on gene expression at the transcriptional level, neglecting the corresponding expression at the protein level can result in unbalanced and potentially biased interpretations of biological effects. Nonetheless, given that non-coding genes vastly outnumber coding genes, this imbalance is likely only a secondary factor contributing to the existence of contradictory genes. Moreover, as previously discussed, substantial evidence supports the notion that variations in pathway expression across distinct tissue environments underscore the presence of contradictory genes. Of particular relevance is the stage-specific expression of these genes. Notably, the overexpression of tumor suppressor genes frequently occurs during the early stages of tumorigenesis, functioning as part of the cellular response to oncogenic stress (Sakaguchi et al., 2003; Ohuchida et al., 2006; Braig et al., 2005; Brambilla et al., 1999). For instance, S100A11 has been identified as a paradoxical gene (Sakaguchi et al., 2003). Sakaguchi et al. demonstrated that S100C/A11 is a critical mediator of calcium-induced growth inhibition in human epidermal keratinocytes by facilitating the phosphorylation-induced nuclear translocation of S100C/A11, thereby halting cell growth (Sakaguchi et al., 2003). However, Ohuchida et al. investigated the expression of the tumor suppressor gene S100A11 across various stages of pancreatic carcinogenesis (Ohuchida et al., 2006). Their research revealed overexpression of S100A11 in the early stages of pancreatic cancer development, such as in intraductal papillary mucinous neoplasms and pancreatic intraepithelial neoplasia (Ohuchida et al., 2006). However, its expression diminishes as the disease progresses advances (Ohuchida et al., 2006). Similarly, the overexpression of the tumor suppressor gene p16INK4a has been documented in early-stage tumors, where it has a crucial role in inducing cellular senescence and halting the proliferation of potentially cancerous cells (Braig et al., 2005; Brambilla et al., 1999). Similar studies not only confirm the existence of paradoxical genes but also emphasize their potential antagonistic effects on tumor progression during tumorigenesis. Paradoxical genes strive to maintain cellular homeostasis. As discussed in our previous work, this antagonistic effect may be linked to alterations in tumor metabolism, TIME, and related signaling pathways, suggesting a potential self-protective mechanism of the body against tumors. We propose that this might represent a form of intrinsic tumor suppression. This overexpression may arise from mechanisms such as subclonal heterogeneity or attempts to balance rapid proliferation and genomic instability. However, once this antagonistic equilibrium is disrupted, paradoxical genes may no longer be able to counteract the progression, leading to their diminished expression and the subsequent unchecked advancement of the tumor towards increased malignancy. Therefore, enhancing the expression of these tumor suppressor genes at early stages could offer promising potential for effective tumor treatment.

In summary, this review introduces the innovative concept of paradoxical genes, highlights the crucial role of tumor suppressor genes in targeted cancer therapy, and provides a theoretical framework for treating cancer by exploiting the balanced interplay between oncogenes and tumor suppressor genes.

## Glossary

ABCA1: ATP-Binding Cassette Transporter A1
ALK: Activin Receptor-Like Kinase
ASB10: Ankyrin Repeat and SOCS Box Containing 10
Bcl-2: B-Cell Lymphoma 2
Bcl-xL: B-Cell Lymphoma-extra Large
BLCA: Bladder Urothelial Carcinoma
BRCA: Breast Cancer
ccRCC: Clear Cell Renal Cell Carcinoma
CDK: Cyclin-Dependent Kinase
CELF2: CUGBP Elav-Like Family Member 2
CHOL: Cholangiocarcinoma
COAD: Colon Adenocarcinoma
CTLs: Cytotoxic T Lymphocytes
CXCL: C-X-C Motif Chemokine Ligand
CXCR: C-X-C Motif Chemokine Receptor
DEA: Differential Expression Analysis
DLL: Delta-Like
DNMAML: Dominant Negative Mastermind-Like
EGFR: Epidermal Growth Factor Receptor
EMT: Epithelial-Mesenchymal Transition
ERK: Extracellular Signal-Regulated Kinase
ESCA: Esophageal Carcinoma
FA: Fatty Acid
FDA: Food and Drug Administration
GO: Gene Ontology
GTEx: Genotype-Tissue Expression
GADD45β: Growth Arrest and DNA Damage-Inducible β
HDAC: Histone Deacetylase
HHLA2: HERV-H LTR-Associating 2
HGP: Human Genome Project
HIF: Hypoxia-Inducible Factor
HNSCC: Head and Neck Squamous Cell Carcinoma
HPV: Human Papillomavirus
IAPs: Inhibitor of Apoptosis Proteins
ICGC: International Cancer Genome Consortium
IRES: Internal Ribosome Entry Sites
IκB: Inhibitor of NF-κB
IPMN: Intraductal Papillary Mucinous Neoplasms
IRE: Inositol-Requiring Enzyme
JNK: c-Jun N-terminal Kinase
KEGG: Kyoto Encyclopedia of Genes and Genomes
KICH: Kidney Chromophobe
KIRC: Kidney Renal Clear Cell Carcinoma
LDLR: Low-Density Lipoprotein Receptor
LIHC: Liver Hepatocellular Carcinoma
LXR: Liver X Receptor
MDSCs: Myeloid-Derived Suppressor Cells
MHC: Major Histocompatibility Complex
NF-κB: Nuclear Factor Kappa Light Chain Enhancer of Activated B Cells
NICD: Notch Intracellular Domain
NK: Natural Killer
NGS: Next-Generation Sequencing
ORF: Open Reading Frame
PD-1: Programmed Cell Death Protein 1
PD-L1: Programmed Death-Ligand 1
PDAC: Pancreatic Ductal Adenocarcinoma
Phox2B: Paired-Like Homeobox 2B
PI3K: Phosphoinositide 3-Kinase
PKCα: Protein Kinase C α
PLK4: Polo-Like Kinase 4
PRAD: Prostate Adenocarcinoma
PTMs: Post-Translational Modifications
QC: Quality Control
RB1: Retinoblastoma 1
RFC4: Replication Factor C Subunit 4
RIDD: Regulated IRE1-Dependent Decay of RNA
SCC: Squamous Cell Carcinoma
SCD1: Stearoyl-CoA Desaturase 1
SNPs: Single Nucleotide Polymorphisms
SREBP-1c: Sterol Regulatory Element-Binding Protein 1c
SUMO: Small Ubiquitin-like Modifier
T-ALL: T-cell Acute Lymphoblastic Leukemia
TCGA: The Cancer Genome Atlas
TGFβ: Transforming Growth Factor β
TEM8: Tumor Endothelial Marker 8
Thr: Threonine
TIME: Tumor Immune Microenvironment
TNBC: Triple-Negative Breast Cancer
Tyr: Tyrosine
uORFs: Upstream Open Reading Frames

## References

[B1] Author anonymous (2012). Comprehensive molecular portraits of human breast tumours. Nature 490 (7418), 61–70. 10.1038/nature11412 23000897 PMC3465532

[B2] Agosto L. M. Mallory M. J. Ferretti M. B. Blake D. Krick K. S. Gazzara M. R. (2023). Alternative splicing of HDAC7 regulates its interaction with 14-3-3 proteins to alter histone marks and target gene expression. Cell Rep. 42 (3), 112273. 10.1016/j.celrep.2023.112273 36933216 PMC10113009

[B3] Ai Q. Ma X. Huang Q. Liu S. Shi T. Zhang C. (2012). High-level expression of Notch1 increased the risk of metastasis in T1 stage clear cell renal cell carcinoma. PLoS One 7 (4), e35022. 10.1371/journal.pone.0035022 22506064 PMC3323638

[B4] Akbani R. Ng P. K. Werner H. M. Shahmoradgoli M. Zhang F. Ju Z. (2014). A pan-cancer proteomic perspective on the Cancer Genome Atlas. Nat. Commun. 5, 3887. 10.1038/ncomms4887 24871328 PMC4109726

[B5] Akhurst R. J. Hata A. (2012). Targeting the TGFβ signalling pathway in disease. Nat. Rev. Drug Discov. 11 (10), 790–811. 10.1038/nrd3810 23000686 PMC3520610

[B6] Al Tarrass M. Belmudes L. Koça D. Azemard V. Liu H. Al T. T. (2024). Large-scale phosphoproteomics reveals activation of the MAPK/GADD45β/P38 axis and cell cycle inhibition in response to BMP9 and BMP10 stimulation in endothelial cells. Cell Commun. Signal 22 (1), 158. 10.1186/s12964-024-01486-0 38439036 PMC10910747

[B7] Ang H. L. Mohan C. D. Shanmugam M. K. Leong H. C. Makvandi P. Rangappa K. S. (2023). Mechanism of epithelial-mesenchymal transition in cancer and its regulation by natural compounds. Med. Res. Rev. 43 (4), 1141–1200. 10.1002/med.21948 36929669

[B8] Annunziata C. M. Davis R. E. Demchenko Y. Bellamy W. Gabrea A. Zhan F. (2007). Frequent engagement of the classical and alternative NF-kappaB pathways by diverse genetic abnormalities in multiple myeloma. Cancer Cell 12 (2), 115–130. 10.1016/j.ccr.2007.07.004 17692804 PMC2730509

[B9] Arima Y. Matsueda S. Saya H. (2023). Significance of cancer-associated fibroblasts in the interactions of cancer cells with the tumor microenvironment of heterogeneous tumor tissue. Cancers (Basel) 15 (9), 2536. 10.3390/cancers15092536 37174001 PMC10177529

[B10] Baker A. Wyatt D. Bocchetta M. Li J. Filipovic A. Green A. (2018). Notch-1-PTEN-ERK1/2 signaling axis promotes HER2+ breast cancer cell proliferation and stem cell survival. Oncogene 37 (33), 4489–4504. 10.1038/s41388-018-0251-y 29743588 PMC9115842

[B11] Baranyi M. Buday L. Hegedűs B. (2020). K-Ras prenylation as a potential anticancer target. Cancer Metastasis Rev. 39 (4), 1127–1141. 10.1007/s10555-020-09902-w 32524209 PMC7680335

[B12] Barash Y. Calarco J. A. Gao W. Pan Q. Wang X. Shai O. (2010). Deciphering the splicing code. Nature 465 (7294), 53–59. 10.1038/nature09000 20445623

[B13] Barbosa C. Peixeiro I. Romão L. (2013). Gene expression regulation by upstream open reading frames and human disease. PLoS Genet. 9 (8), e1003529. 10.1371/journal.pgen.1003529 23950723 PMC3738444

[B14] Bardelli V. Arniani S. Pierini V. Di Giacomo D. Pierini T. Gorello P. (2021). T-cell acute lymphoblastic leukemia: biomarkers and their clinical usefulness. Genes (Basel) 12 (8), 1118. 10.3390/genes12081118 34440292 PMC8394887

[B15] Baud V. Karin M. (2009). Is NF-kappaB a good target for cancer therapy? Hopes and pitfalls. Nat. Rev. Drug Discov. 8 (1), 33–40. 10.1038/nrd2781 19116625 PMC2729321

[B16] Becker K. F. Atkinson M. J. Reich U. Becker I. Nekarda H. Siewert J. R. (1994). E-cadherin gene mutations provide clues to diffuse type gastric carcinomas. Cancer Res. 54 (14), 3845–3852. 8033105

[B17] Bellavia D. Mecarozzi M. Campese A. F. Grazioli P. Gulino A. Screpanti I. (2007). Notch and Ikaros: not only converging players in T cell leukemia. Cell Cycle 6 (22), 2730–2734. 10.4161/cc.6.22.4894 18032925

[B18] Bertrand F. E. Angus C. W. Partis W. J. Sigounas G. (2012). Developmental pathways in colon cancer: crosstalk between WNT, BMP, Hedgehog and Notch. Cell Cycle 11 (23), 4344–4351. 10.4161/cc.22134 23032367 PMC3552917

[B19] Beyer A. Bandyopadhyay S. Ideker T. (2007). Integrating physical and genetic maps: from genomes to interaction networks. Nat. Rev. Genet. 8 (9), 699–710. 10.1038/nrg2144 17703239 PMC2811081

[B20] Bishop J. M. (1991). Molecular themes in oncogenesis. Cell 64 (2), 235–248. 10.1016/0092-8674(91)90636-d 1988146

[B21] Black D. L. (2003). Mechanisms of alternative pre-messenger RNA splicing. Annu. Rev. Biochem. 72, 291–336. 10.1146/annurev.biochem.72.121801.161720 12626338

[B22] Blagih J. Buck M. D. Vousden K. H. (2020). p53, cancer and the immune response. J. Cell Sci. 133 (5), jcs237453. 10.1242/jcs.237453 32144194

[B23] Boor P. P. C. Sideras K. Biermann K. Hosein Aziz M. Levink I. J. M. Mancham S. (2020). HHLA2 is expressed in pancreatic and ampullary cancers and increased expression is associated with better post-surgical prognosis. Br. J. Cancer 122 (8), 1211–1218. 10.1038/s41416-020-0755-4 32071413 PMC7156757

[B24] Braig M. Lee S. Loddenkemper C. Rudolph C. Peters A. H. Schlegelberger B. (2005). Oncogene-induced senescence as an initial barrier in lymphoma development. Nature 436 (7051), 660–665. 10.1038/nature03841 16079837

[B25] Brambilla E. Gazzeri S. Moro D. Lantuejoul S. Veyrenc S. Brambilla C. (1999). Alterations of Rb pathway (Rb-p16INK4-cyclin D1) in preinvasive bronchial lesions. Clin. Cancer Res. 5 (2), 243–250. 10037171

[B26] Bray S. J. (2016). Notch signalling in context. Nat. Rev. Mol. Cell Biol. 17 (11), 722–735. 10.1038/nrm.2016.94 27507209

[B27] Brown J. M. Wilson W. R. (2004). Exploiting tumour hypoxia in cancer treatment. Nat. Rev. Cancer 4 (6), 437–447. 10.1038/nrc1367 15170446

[B28] Brzozowa-Zasada M. (2022). Prognostic significance of Notch3 immunoreactivity patterns in Caucasian colon adenocarcinoma patients. Prz. Gastroenterol. 17 (2), 162–168. 10.5114/pg.2022.116389 35664020 PMC9165326

[B29] Caliskan N. Peske F. Rodnina M. V. (2015). Changed in translation: mRNA recoding by -1 programmed ribosomal frameshifting. Trends Biochem. Sci. 40 (5), 265–274. 10.1016/j.tibs.2015.03.006 25850333 PMC7126180

[B30] Calvo S. E. Pagliarini D. J. Mootha V. K. (2009). Upstream open reading frames cause widespread reduction of protein expression and are polymorphic among humans. Proc. Natl. Acad. Sci. U. S. A. 106 (18), 7507–7512. 10.1073/pnas.0810916106 19372376 PMC2669787

[B31] Cancer Genome Atlas Research Network (2008). Comprehensive genomic characterization defines human glioblastoma genes and core pathways. Nature 455 (7216), 1061–1068. 10.1038/nature07385 18772890 PMC2671642

[B32] Cantley L. C. Neel B. G. (1999). New insights into tumor suppression: PTEN suppresses tumor formation by restraining the phosphoinositide 3-kinase/AKT pathway. Proc. Natl. Acad. Sci. U. S. A. 96 (8), 4240–4245. 10.1073/pnas.96.8.4240 10200246 PMC33561

[B33] Cao Y. Jiao N. Sun T. Ma Y. Zhang X. Chen H. (2021). CXCL11 correlates with antitumor immunity and an improved prognosis in colon cancer. Front. Cell Dev. Biol. 9, 646252. 10.3389/fcell.2021.646252 33777950 PMC7991085

[B34] Carvalho F. L. Simons B. W. Eberhart C. G. Berman D. M. (2014). Notch signaling in prostate cancer: a moving target. Prostate 74 (9), 933–945. 10.1002/pros.22811 24737393 PMC4323172

[B35] Cascio S. D'Andrea A. Ferla R. Surmacz E. Gulotta E. Amodeo V. (2010). miR-20b modulates VEGF expression by targeting HIF-1 alpha and STAT3 in MCF-7 breast cancer cells. J. Cell Physiol. 224 (1), 242–249. 10.1002/jcp.22126 20232316

[B36] Chang H. M. Yeh E. T. H. (2020). SUMO: from bench to bedside. Physiol. Rev. 100 (4), 1599–1619. 10.1152/physrev.00025.2019 32666886 PMC7717128

[B37] Chawla A. Repa J. J. Evans R. M. Mangelsdorf D. J. (2001). Nuclear receptors and lipid physiology: opening the X-files. Science. 294 (5548), 1866–1870. 10.1126/science.294.5548.1866 11729302

[B38] Chen D. Li M. Luo J. Gu W. (2003). Direct interactions between HIF-1 alpha and Mdm2 modulate p53 function. J. Biol. Chem. 278 (16), 13595–13598. 10.1074/jbc.C200694200 12606552

[B39] Chen M. Manley J. L. (2009). Mechanisms of alternative splicing regulation: insights from molecular and genomics approaches. Nat. Rev. Mol. Cell Biol. 10 (11), 741–754. 10.1038/nrm2777 19773805 PMC2958924

[B40] Ciechanover A. (2005). Proteolysis: from the lysosome to ubiquitin and the proteasome. Nat. Rev. Mol. Cell Biol. 6 (1), 79–87. 10.1038/nrm1552 15688069

[B41] Ciechanover A. Kwon Y. T. (2015). Degradation of misfolded proteins in neurodegenerative diseases: therapeutic targets and strategies. Exp. Mol. Med. 47 (3), e147. 10.1038/emm.2014.117 25766616 PMC4351408

[B42] Cieśla M. Ngoc P. C. T. Muthukumar S. Todisco G. Madej M. Fritz H. (2023). m(6)A-driven SF3B1 translation control steers splicing to direct genome integrity and leukemogenesis. Mol. Cell 83 (7), 1165–1179.e11. 10.1016/j.molcel.2023.02.024 36944332

[B43] Cohen P. (2000). The regulation of protein function by multisite phosphorylation--a 25 year update. Trends Biochem. Sci. 25 (12), 596–601. 10.1016/s0968-0004(00)01712-6 11116185

[B44] Colak S. Ten Dijke P. (2017). Targeting TGF-β signaling in cancer. Trends Cancer 3 (1), 56–71. 10.1016/j.trecan.2016.11.008 28718426

[B45] Coussens L. M. Werb Z. (2002). Inflammation and cancer. Nature 420 (6917), 860–867. 10.1038/nature01322 12490959 PMC2803035

[B46] Crick F. (1970). Central dogma of molecular biology. Nature 227 (5258), 561–563. 10.1038/227561a0 4913914

[B47] Croce C. M. (2008). Oncogenes and cancer. N. Engl. J. Med. 358 (5), 502–511. 10.1056/NEJMra072367 18234754

[B48] Damaghi M. Wojtkowiak J. W. Gillies R. J. (2013). pH sensing and regulation in cancer. Front. Physiol. 4, 370. 10.3389/fphys.2013.00370 24381558 PMC3865727

[B49] David C. J. Manley J. L. (2010). Alternative pre-mRNA splicing regulation in cancer: pathways and programs unhinged. Genes Dev. 24 (21), 2343–2364. 10.1101/gad.1973010 21041405 PMC2964746

[B50] Dawson M. A. Kouzarides T. (2012). Cancer epigenetics: from mechanism to therapy. Cell 150 (1), 12–27. 10.1016/j.cell.2012.06.013 22770212

[B51] Decker J. T. Ma J. A. Shea L. D. Jeruss J. S. (2021). Implications of TGFβ signaling and CDK inhibition for the treatment of breast cancer. Cancers (Basel) 13 (21), 5343. 10.3390/cancers13215343 34771508 PMC8582459

[B52] Derynck R. (1994). TGF-beta-receptor-mediated signaling. Trends Biochem. Sci. 19 (12), 548–553. 10.1016/0968-0004(94)90059-0 7846768

[B53] Derynck R. Akhurst R. J. (2007). Differentiation plasticity regulated by TGF-beta family proteins in development and disease. Nat. Cell Biol. 9 (9), 1000–1004. 10.1038/ncb434 17762890

[B54] Derynck R. Zhang Y. E. (2003). Smad-dependent and Smad-independent pathways in TGF-beta family signalling. Nature 425 (6958), 577–584. 10.1038/nature02006 14534577

[B55] Deshaies R. J. Ferrell J. E. Jr (2001). Multisite phosphorylation and the countdown to S phase. Cell 107 (7), 819–822. 10.1016/s0092-8674(01)00620-1 11779457

[B56] De Smaele E. Zazzeroni F. Papa S. Nguyen D. U. Jin R. Jones J. (2001). Induction of gadd45beta by NF-kappaB downregulates pro-apoptotic JNK signalling. Nature 414 (6861), 308–313. 10.1038/35104560 11713530

[B57] de Visser K. E. Eichten A. Coussens L. M. (2006). Paradoxical roles of the immune system during cancer development. Nat. Rev. Cancer 6 (1), 24–37. 10.1038/nrc1782 16397525

[B58] Drazic A. Myklebust L. M. Ree R. Arnesen T. (2016). The world of protein acetylation. Biochim. Biophys. Acta 1864 (10), 1372–1401. 10.1016/j.bbapap.2016.06.007 27296530

[B59] Duan G. Walther D. (2015). The roles of post-translational modifications in the context of protein interaction networks. PLoS Comput. Biol. 11 (2), e1004049. 10.1371/journal.pcbi.1004049 25692714 PMC4333291

[B60] Ellisen L. W. Bird J. West D. C. Soreng A. L. Reynolds T. C. Smith S. D. (1991). TAN-1, the human homolog of the Drosophila notch gene, is broken by chromosomal translocations in T lymphoblastic neoplasms. Cell 66 (4), 649–661. 10.1016/0092-8674(91)90111-b 1831692

[B61] Feil R. Fraga M. F. (2012). Epigenetics and the environment: emerging patterns and implications. Nat. Rev. Genet. 13 (2), 97–109. 10.1038/nrg3142 22215131

[B62] Fessler M. B. (2016). The intracellular cholesterol landscape: dynamic integrator of the immune response. Trends Immunol. 37 (12), 819–830. 10.1016/j.it.2016.09.001 27692616 PMC5135597

[B63] Flotho A. Melchior F. (2013). Sumoylation: a regulatory protein modification in health and disease. Annu. Rev. Biochem. 82, 357–385. 10.1146/annurev-biochem-061909-093311 23746258

[B64] Fortini M. E. (2009). Notch signaling: the core pathway and its posttranslational regulation. Dev. Cell 16 (5), 633–647. 10.1016/j.devcel.2009.03.010 19460341

[B65] Fridman W. H. Pagès F. Sautès-Fridman C. Galon J. (2012). The immune contexture in human tumours: impact on clinical outcome. Nat. Rev. Cancer 12 (4), 298–306. 10.1038/nrc3245 22419253

[B66] Fukuchi J. Kokontis J. M. Hiipakka R. A. Chuu C. P. Liao S. (2004). Antiproliferative effect of liver X receptor agonists on LNCaP human prostate cancer cells. Cancer Res. 64 (21), 7686–7689. 10.1158/0008-5472.CAN-04-2332 15520170

[B67] Garcia-Carbonero N. Li W. Cabeza-Morales M. Martinez-Useros J. Garcia-Foncillas J. (2018). New hope for pancreatic ductal adenocarcinoma treatment targeting endoplasmic reticulum stress response: a systematic review. Int. J. Mol. Sci. 19 (9), 2468. 10.3390/ijms19092468 30134550 PMC6165247

[B68] Gatenby R. A. Gillies R. J. (2004). Why do cancers have high aerobic glycolysis? Nat. Rev. Cancer 4 (11), 891–899. 10.1038/nrc1478 15516961

[B69] Geiss-Friedlander R. Melchior F. (2007). Concepts in sumoylation: a decade on. Nat. Rev. Mol. Cell Biol. 8 (12), 947–956. 10.1038/nrm2293 18000527

[B70] Gerlinger M. Rowan A. J. Horswell S. Math M. Larkin J. Endesfelder D. (2012). Intratumor heterogeneity and branched evolution revealed by multiregion sequencing. N. Engl. J. Med. 366 (10), 883–892. 10.1056/NEJMoa1113205 22397650 PMC4878653

[B71] Gillies R. J. Gatenby R. A. (2007). Hypoxia and adaptive landscapes in the evolution of carcinogenesis. Cancer Metastasis Rev. 26 (2), 311–317. 10.1007/s10555-007-9065-z 17404691

[B72] Gillies R. J. Robey I. Gatenby R. A. (2008). Causes and consequences of increased glucose metabolism of cancers. J. Nucl. Med. 49 (Suppl. 2), 24S-42S–42s. 10.2967/jnumed.107.047258 18523064

[B73] Gilmore T. D. Herscovitch M. (2006). Inhibitors of NF-kappaB signaling: 785 and counting. Oncogene 25 (51), 6887–6899. 10.1038/sj.onc.1209982 17072334

[B74] Giovannini C. Bolondi L. Gramantieri L. (2016). Targeting Notch3 in hepatocellular carcinoma: molecular mechanisms and therapeutic perspectives. Int. J. Mol. Sci. 18 (1), 56. 10.3390/ijms18010056 28036048 PMC5297691

[B75] Giovannini C. Minguzzi M. Baglioni M. Fornari F. Giannone F. Ravaioli M. (2014). Suppression of p53 by Notch3 is mediated by Cyclin G1 and sustained by MDM2 and miR-221 axis in hepatocellular carcinoma. Oncotarget 5 (21), 10607–10620. 10.18632/oncotarget.2523 25431954 PMC4279397

[B76] Golub T. R. Slonim D. K. Tamayo P. Huard C. Gaasenbeek M. Mesirov J. P. (1999). Molecular classification of cancer: class discovery and class prediction by gene expression monitoring. Science 286 (5439), 531–537. 10.1126/science.286.5439.531 10521349

[B77] Gottesman M. M. (2002). Mechanisms of cancer drug resistance. Annu. Rev. Med. 53, 615–627. 10.1146/annurev.med.53.082901.103929 11818492

[B78] Greer E. L. Shi Y. (2012). Histone methylation: a dynamic mark in health, disease and inheritance. Nat. Rev. Genet. 13 (5), 343–357. 10.1038/nrg3173 22473383 PMC4073795

[B79] Greten F. R. Eckmann L. Greten T. F. Park J. M. Li Z. W. Egan L. J. (2004). IKKbeta links inflammation and tumorigenesis in a mouse model of colitis-associated cancer. Cell 118 (3), 285–296. 10.1016/j.cell.2004.07.013 15294155

[B80] Greten F. R. Karin M. (2004). The IKK/NF-kappaB activation pathway-a target for prevention and treatment of cancer. Cancer Lett. 206 (2), 193–199. 10.1016/j.canlet.2003.08.029 15013524

[B81] Grivennikov S. I. Greten F. R. Karin M. (2010). Immunity, inflammation, and cancer. Cell 140 (6), 883–899. 10.1016/j.cell.2010.01.025 20303878 PMC2866629

[B82] Han N. Yuan M. Yan L. Tang H. (2023). Emerging insights into liver X receptor α in the tumorigenesis and therapeutics of human cancers. Biomolecules 13 (8), 1184. 10.3390/biom13081184 37627249 PMC10452869

[B83] Han Q. Han F. Fan Y. Lian B. Xiao J. Sun W. (2020). Notch3 is involved in the proliferation of renal cancer cells via regulation of cell cycle progression and HIF-2α. Oncol. Lett. 20 (6), 379. 10.3892/ol.2020.12242 33154777 PMC7608028

[B84] Hanahan D. Weinberg R. A. (2000). The hallmarks of cancer. Cell 100 (1), 57–70. 10.1016/s0092-8674(00)81683-9 10647931

[B85] Hanahan D. Weinberg R. A. (2011). Hallmarks of cancer: the next generation. Cell 144 (5), 646–674. 10.1016/j.cell.2011.02.013 21376230

[B86] Hao Y. Baker D. Ten Dijke P. (2019). TGF-β-Mediated epithelial-mesenchymal transition and cancer metastasis. Int. J. Mol. Sci. 20 (11), 2767. 10.3390/ijms20112767 31195692 PMC6600375

[B87] Harnoss J. M. Le Thomas A. Reichelt M. Guttman O. Wu T. D. Marsters S. A. (2020). IRE1α disruption in triple-negative breast cancer cooperates with antiangiogenic therapy by reversing ER stress adaptation and remodeling the tumor microenvironment. Cancer Res. 80 (11), 2368–2379. 10.1158/0008-5472.CAN-19-3108 32265225 PMC7272310

[B88] Harris A. L. (2002). Hypoxia--a key regulatory factor in tumour growth. Nat. Rev. Cancer 2 (1), 38–47. 10.1038/nrc704 11902584

[B89] He W. Li Q. Li X. (2023). Acetyl-CoA regulates lipid metabolism and histone acetylation modification in cancer. Biochim. Biophys. Acta Rev. Cancer 1878 (1), 188837. 10.1016/j.bbcan.2022.188837 36403921

[B90] Herbertz S. Sawyer J. S. Stauber A. J. Gueorguieva I. Driscoll K. E. Estrem S. T. (2015). Clinical development of galunisertib (LY2157299 monohydrate), a small molecule inhibitor of transforming growth factor-beta signaling pathway. Drug Des. Devel Ther. 9, 4479–4499. 10.2147/DDDT.S86621 PMC4539082 26309397

[B91] Hershko A. Ciechanover A. (1998). The ubiquitin system. Annu. Rev. Biochem. 67, 425–479. 10.1146/annurev.biochem.67.1.425 9759494

[B92] Hinnebusch A. G. Ivanov I. P. Sonenberg N. (2016). Translational control by 5'-untranslated regions of eukaryotic mRNAs. Science 352 (6292), 1413–1416. 10.1126/science.aad9868 27313038 PMC7422601

[B93] Ho P. C. Bihuniak J. D. Macintyre A. N. Staron M. Liu X. Amezquita R. (2015). Phosphoenolpyruvate is a metabolic checkpoint of anti-tumor T cell responses. Cell 162 (6), 1217–1228. 10.1016/j.cell.2015.08.012 26321681 PMC4567953

[B94] Huang S. (2009). Reprogramming cell fates: reconciling rarity with robustness. Bioessays. 31 (5), 546–560. 10.1002/bies.200800189 19319911

[B95] Hunter T. (2007). The age of crosstalk: phosphorylation, ubiquitination, and beyond. Mol. Cell 28 (5), 730–738. 10.1016/j.molcel.2007.11.019 18082598

[B96] Inman G. J. Nicolás F. J. Callahan J. F. Harling J. D. Gaster L. M. Reith A. D. (2002). SB-431542 is a potent and specific inhibitor of transforming growth factor-beta superfamily type I activin receptor-like kinase (ALK) receptors ALK4, ALK5, and ALK7. Mol. Pharmacol. 62 (1), 65–74. 10.1124/mol.62.1.65 12065756

[B97] Ishiguro H. Okubo T. Kuwabara Y. Kimura M. Mitsui A. Sugito N. (2017). NOTCH1 activates the Wnt/β-catenin signaling pathway in colon cancer. Oncotarget 8 (36), 60378–60389. 10.18632/oncotarget.19534 28947978 PMC5601146

[B98] Ivanov I. P. Loughran G. Sachs M. S. Atkins J. F. (2010). Initiation context modulates autoregulation of eukaryotic translation initiation factor 1 (eIF1). Proc. Natl. Acad. Sci. U. S. A. 107 (42), 18056–18060. 10.1073/pnas.1009269107 20921384 PMC2964218

[B99] Jackson R. J. Hellen C. U. Pestova T. V. (2010). The mechanism of eukaryotic translation initiation and principles of its regulation. Nat. Rev. Mol. Cell Biol. 11 (2), 113–127. 10.1038/nrm2838 20094052 PMC4461372

[B100] Janku F. Yap T. A. Meric-Bernstam F. (2018). Targeting the PI3K pathway in cancer: are we making headway? Nat. Rev. Clin. Oncol. 15 (5), 273–291. 10.1038/nrclinonc.2018.28 29508857

[B101] Janssens S. Tschopp J. (2006). Signals from within: the DNA-damage-induced NF-kappaB response. Cell Death Differ. 13 (5), 773–784. 10.1038/sj.cdd.4401843 16410802

[B102] Jarome T. J. Butler A. A. Nichols J. N. Pacheco N. L. Lubin F. D. (2015). NF-κB mediates Gadd45β expression and DNA demethylation in the hippocampus during fear memory formation. Front. Mol. Neurosci. 8, 54. 10.3389/fnmol.2015.00054 26441517 PMC4584956

[B103] Jeong D. W. Lee S. Chun Y. S. (2021). How cancer cells remodel lipid metabolism: strategies targeting transcription factors. Lipids Health Dis. 20 (1), 163. 10.1186/s12944-021-01593-8 34775964 PMC8590761

[B104] Johnstone T. G. Bazzini A. A. Giraldez A. J. (2016). Upstream ORFs are prevalent translational repressors in vertebrates. Embo J. 35 (7), 706–723. 10.15252/embj.201592759 26896445 PMC4818764

[B105] Joseph S. B. Castrillo A. Laffitte B. A. Mangelsdorf D. J. Tontonoz P. (2003). Reciprocal regulation of inflammation and lipid metabolism by liver X receptors. Nat. Med. 9 (2), 213–219. 10.1038/nm820 12524534

[B106] Joyce J. A. Fearon D. T. (2015). T cell exclusion, immune privilege, and the tumor microenvironment. Science. 348 (6230), 74–80. 10.1126/science.aaa6204 25838376

[B107] Jung D. Bachmann H. S. (2023). Regulation of protein prenylation. Biomed. Pharmacother. 164, 114915. 10.1016/j.biopha.2023.114915 37236024

[B108] Kaelin W. G. Jr (1997). Alterations in G1/S cell-cycle control contributing to carcinogenesis. Ann. N. Y. Acad. Sci. 833, 29–33. 10.1111/j.1749-6632.1997.tb48589.x 9616737

[B109] Kałafut J. Czerwonka A. Anameriç A. Przybyszewska-Podstawka A. Misiorek J. O. Rivero-Müller A. (2021). Shooting at moving and hidden targets-tumour cell plasticity and the notch signalling pathway in head and neck squamous cell carcinomas. Cancers (Basel) 13 (24), 6219. 10.3390/cancers13246219 34944837 PMC8699303

[B110] Kalluri R. Weinberg R. A. (2009). The basics of epithelial-mesenchymal transition. J. Clin. Invest 119 (6), 1420–1428. 10.1172/JCI39104 19487818 PMC2689101

[B111] Kalsotra A. Cooper T. A. (2011). Functional consequences of developmentally regulated alternative splicing. Nat. Rev. Genet. 12 (10), 715–729. 10.1038/nrg3052 21921927 PMC3321218

[B112] Karin M. (2006). Nuclear factor-kappaB in cancer development and progression. Nature 441 (7092), 431–436. 10.1038/nature04870 16724054

[B113] Karin M. Cao Y. Greten F. R. Li Z. W. (2002). NF-kappaB in cancer: from innocent bystander to major culprit. Nat. Rev. Cancer 2 (4), 301–310. 10.1038/nrc780 12001991

[B114] Karin M. Greten F. R. (2005). NF-kappaB: linking inflammation and immunity to cancer development and progression. Nat. Rev. Immunol. 5 (10), 749–759. 10.1038/nri1703 16175180

[B115] Kawaguchi K. Kaneko S. (2021). Notch signaling and liver cancer. Adv. Exp. Med. Biol. 1287, 69–80. 10.1007/978-3-030-55031-8_6 33034027

[B116] Keith B. Johnson R. S. Simon M. C. (2011). HIF1α and HIF2α: sibling rivalry in hypoxic tumour growth and progression. Nat. Rev. Cancer 12 (1), 9–22. 10.1038/nrc3183 22169972 PMC3401912

[B117] Kim W. Khan S. K. Yang Y. (2017). Interacting network of Hippo, Wnt/β-catenin and Notch signaling represses liver tumor formation. BMB Rep. 50 (1), 1–2. 10.5483/bmbrep.2017.50.1.196 27881216 PMC5319656

[B118] Klose R. J. Zhang Y. (2007). Regulation of histone methylation by demethylimination and demethylation. Nat. Rev. Mol. Cell Biol. 8 (4), 307–318. 10.1038/nrm2143 17342184

[B119] Kopan R. Ilagan M. X. (2009). The canonical Notch signaling pathway: unfolding the activation mechanism. Cell 137 (2), 216–233. 10.1016/j.cell.2009.03.045 19379690 PMC2827930

[B120] Kornblihtt A. R. Schor I. E. Alló M. Dujardin G. Petrillo E. Muñoz M. J. (2013). Alternative splicing: a pivotal step between eukaryotic transcription and translation. Nat. Rev. Mol. Cell Biol. 14 (3), 153–165. 10.1038/nrm3525 23385723

[B121] Kosti I. Jain N. Aran D. Butte A. J. Sirota M. (2016). Cross-tissue analysis of gene and protein expression in normal and cancer tissues. Sci. Rep. 6, 24799. 10.1038/srep24799 27142790 PMC4855174

[B122] Kozak M. (1987). Effects of intercistronic length on the efficiency of reinitiation by eucaryotic ribosomes. Mol. Cell Biol. 7 (10), 3438–3445. 10.1128/mcb.7.10.3438 3683388 PMC367994

[B123] Kozak M. (1999). Initiation of translation in prokaryotes and eukaryotes. Gene 234 (2), 187–208. 10.1016/s0378-1119(99)00210-3 10395892

[B124] Kozak M. (2001). Constraints on reinitiation of translation in mammals. Nucleic Acids Res. 29 (24), 5226–5232. 10.1093/nar/29.24.5226 11812856 PMC97554

[B125] Kozak M. (2005). Regulation of translation via mRNA structure in prokaryotes and eukaryotes. Gene 361, 13–37. 10.1016/j.gene.2005.06.037 16213112

[B126] Krieg A. J. Rankin E. B. Chan D. Razorenova O. Fernandez S. Giaccia A. J. (2010). Regulation of the histone demethylase JMJD1A by hypoxia-inducible factor 1 alpha enhances hypoxic gene expression and tumor growth. Mol. Cell Biol. 30 (1), 344–353. 10.1128/MCB.00444-09 19858293 PMC2798291

[B127] Kulis M. Esteller M. (2010). DNA methylation and cancer. Adv. Genet. 70, 27–56. 10.1016/B978-0-12-380866-0.60002-2 20920744

[B128] Kulshreshtha R. Ferracin M. Wojcik S. E. Garzon R. Alder H. Agosto-Perez F. J. (2007). A microRNA signature of hypoxia. Mol. Cell Biol. 27 (5), 1859–1867. 10.1128/MCB.01395-06 17194750 PMC1820461

[B129] Lee G. H. Yoo K. C. An Y. Lee H. J. Lee M. Uddin N. (2018). FYN promotes mesenchymal phenotypes of basal type breast cancer cells through STAT5/NOTCH2 signaling node. Oncogene 37 (14), 1857–1868. 10.1038/s41388-017-0114-y 29348460

[B130] Leontovich A. A. Jalalirad M. Salisbury J. L. Mills L. Haddox C. Schroeder M. (2018). NOTCH3 expression is linked to breast cancer seeding and distant metastasis. Breast Cancer Res. 20 (1), 105. 10.1186/s13058-018-1020-0 30180881 PMC6123953

[B131] Li J. Yen C. Liaw D. Podsypanina K. Bose S. Wang S. I. (1997). PTEN, a putative protein tyrosine phosphatase gene mutated in human brain, breast, and prostate cancer. Science. 275 (5308), 1943–1947. 10.1126/science.275.5308.1943 9072974

[B132] Li T. Wen H. Brayton C. Das P. Smithson L. A. Fauq A. (2007). Epidermal growth factor receptor and notch pathways participate in the tumor suppressor function of gamma-secretase. J. Biol. Chem. 282 (44), 32264–32273. 10.1074/jbc.M703649200 17827153

[B133] Lim S. O. Li C. W. Xia W. Cha J. H. Chan L. C. Wu Y. (2016). Deubiquitination and stabilization of PD-L1 by CSN5. Cancer Cell 30 (6), 925–939. 10.1016/j.ccell.2016.10.010 27866850 PMC5171205

[B134] Liu L. Tao T. Liu S. Yang X. Chen X. Liang J. (2021b). An RFC4/Notch1 signaling feedback loop promotes NSCLC metastasis and stemness. Nat. Commun. 12 (1), 2693. 10.1038/s41467-021-22971-x 33976158 PMC8113560

[B135] Liu Y. Beyer A. Aebersold R. (2016). On the dependency of cellular protein levels on mRNA abundance. Cell 165 (3), 535–550. 10.1016/j.cell.2016.03.014 27104977

[B136] Liu Y. Teng L. Fu S. Wang G. Li Z. Ding C. (2021a). Highly heterogeneous-related genes of triple-negative breast cancer: potential diagnostic and prognostic biomarkers. BMC Cancer 21 (1), 644. 10.1186/s12885-021-08318-1 34053447 PMC8165798

[B137] Manning G. Whyte D. B. Martinez R. Hunter T. Sudarsanam S. (2002). The protein kinase complement of the human genome. Science. 298 (5600), 1912–1934. 10.1126/science.1075762 12471243

[B138] Martinez-Turtos A. Paul R. Grima-Reyes M. Issaoui H. Krug A. Mhaidly R. (2022). IRE1α overexpression in malignant cells limits tumor progression by inducing an anti-cancer immune response. Oncoimmunology 11 (1), 2116844. 10.1080/2162402X.2022.2116844 36046811 PMC9423862

[B139] Marusyk A. Almendro V. Polyak K. (2012). Intra-tumour heterogeneity: a looking glass for cancer? Nat. Rev. Cancer 12 (5), 323–334. 10.1038/nrc3261 22513401

[B140] Massagué J. (2008). TGFbeta in cancer. Cell. 134 (2), 215–230. 10.1016/j.cell.2008.07.001 18662538 PMC3512574

[B141] McGillivray P. Ault R. Pawashe M. Kitchen R. Balasubramanian S. Gerstein M. (2018). A comprehensive catalog of predicted functional upstream open reading frames in humans. Nucleic Acids Res. 46 (7), 3326–3338. 10.1093/nar/gky188 29562350 PMC6283423

[B142] Mellman I. Coukos G. Dranoff G. (2011). Cancer immunotherapy comes of age. Nature 480 (7378), 480–489. 10.1038/nature10673 22193102 PMC3967235

[B143] Menendez J. A. Lupu R. (2007). Fatty acid synthase and the lipogenic phenotype in cancer pathogenesis. Nat. Rev. Cancer 7 (10), 763–777. 10.1038/nrc2222 17882277

[B144] Meng F. Henson R. Wehbe-Janek H. Ghoshal K. Jacob S. T. Patel T. (2007). MicroRNA-21 regulates expression of the PTEN tumor suppressor gene in human hepatocellular cancer. Gastroenterology 133 (2), 647–658. 10.1053/j.gastro.2007.05.022 17681183 PMC4285346

[B145] Moreau P. Avet-Loiseau H. Facon T. Attal M. Tiab M. Hulin C. (2011). Bortezomib plus dexamethasone versus reduced-dose bortezomib, thalidomide plus dexamethasone as induction treatment before autologous stem cell transplantation in newly diagnosed multiple myeloma. Blood 118 (22), 5752–5758. quiz 982. 10.1182/blood-2011-05-355081 21849487

[B146] Morris D. R. Geballe A. P. (2000). Upstream open reading frames as regulators of mRNA translation. Mol. Cell Biol. 20 (23), 8635–8642. 10.1128/mcb.20.23.8635-8642.2000 11073965 PMC86464

[B147] Mortazavi A. Williams B. A. McCue K. Schaeffer L. Wold B. (2008). Mapping and quantifying mammalian transcriptomes by RNA-Seq. Nat. Methods 5 (7), 621–628. 10.1038/nmeth.1226 18516045 PMC13303166

[B148] Müller I. Munder M. Kropf P. Hänsch G. M. (2009). Polymorphonuclear neutrophils and T lymphocytes: strange bedfellows or brothers in arms? Trends Immunol. 30 (11), 522–530. 10.1016/j.it.2009.07.007 19775938

[B149] Nadal E. Saleh M. Aix S. P. Ochoa-de-Olza M. Patel S. P. Antonia S. (2023). A phase Ib/II study of galunisertib in combination with nivolumab in solid tumors and non-small cell lung cancer. BMC Cancer 23 (1), 708. 10.1186/s12885-023-11153-1 37507657 PMC10386782

[B150] Nelson B. H. (2010). CD20+ B cells: the other tumor-infiltrating lymphocytes. J. Immunol. 185 (9), 4977–4982. 10.4049/jimmunol.1001323 20962266

[B151] Nguyen-Vu T. Vedin L. L. Liu K. Jonsson P. Lin J. Z. Candelaria N. R. (2013). Liver × receptor ligands disrupt breast cancer cell proliferation through an E2F-mediated mechanism. Breast Cancer Res. 15 (3), R51. 10.1186/bcr3443 23809258 PMC4053202

[B152] Nilsen T. W. Graveley B. R. (2010). Expansion of the eukaryotic proteome by alternative splicing. Nature 463 (7280), 457–463. 10.1038/nature08909 20110989 PMC3443858

[B153] Nishi H. Shaytan A. Panchenko A. R. (2014). Physicochemical mechanisms of protein regulation by phosphorylation. Front. Genet. 5, 270. 10.3389/fgene.2014.00270 25147561 PMC4124799

[B154] Niu X. Ren L. Hu A. Zhang S. Qi H. (2022). Identification of potential diagnostic and prognostic biomarkers for gastric cancer based on bioinformatic analysis. Front. Genet. 13, 862105. 10.3389/fgene.2022.862105 35368700 PMC8966486

[B155] Oeckinghaus A. Hayden M. S. Ghosh S. (2011). Crosstalk in NF-κB signaling pathways. Nat. Immunol. 12 (8), 695–708. 10.1038/ni.2065 21772278

[B156] Ohuchida K. Mizumoto K. Ohhashi S. Yamaguchi H. Konomi H. Nagai E. (2006). S100A11, a putative tumor suppressor gene, is overexpressed in pancreatic carcinogenesis. Clin. Cancer Res. 12 (18), 5417–5422. 10.1158/1078-0432.CCR-06-0222 17000675

[B157] Okazaki H. Goldstein J. L. Brown M. S. Liang G. (2010). LXR-SREBP-1c-phospholipid transfer protein axis controls very low density lipoprotein (VLDL) particle size. J. Biol. Chem. 285 (9), 6801–6810. 10.1074/jbc.M109.079459 20037162 PMC2825474

[B158] Olsen J. V. Blagoev B. Gnad F. Macek B. Kumar C. Mortensen P. (2006). Global, *in vivo*, and site-specific phosphorylation dynamics in signaling networks. Cell 127 (3), 635–648. 10.1016/j.cell.2006.09.026 17081983

[B159] Ozsolak F. Milos P. M. (2011). RNA sequencing: advances, challenges and opportunities. Nat. Rev. Genet. 12 (2), 87–98. 10.1038/nrg2934 21191423 PMC3031867

[B160] Padmanaban V. Krol I. Suhail Y. Szczerba B. M. Aceto N. Bader J. S. (2019). E-cadherin is required for metastasis in multiple models of breast cancer. Nature 573 (7774), 439–444. 10.1038/s41586-019-1526-3 31485072 PMC7365572

[B161] Palucka K. Banchereau J. (2012). Cancer immunotherapy via dendritic cells. Nat. Rev. Cancer 12 (4), 265–277. 10.1038/nrc3258 22437871 PMC3433802

[B162] Pancewicz-Wojtkiewicz J. (2016). Epidermal growth factor receptor and notch signaling in non-small-cell lung cancer. Cancer Med. 5 (12), 3572–3578. 10.1002/cam4.944 27770511 PMC5224843

[B163] Pandya K. Wyatt D. Gallagher B. Shah D. Baker A. Bloodworth J. (2016). PKCα attenuates jagged-1-mediated notch signaling in ErbB-2-positive breast cancer to reverse trastuzumab resistance. Clin. Cancer Res. 22 (1), 175–186. 10.1158/1078-0432.CCR-15-0179 26350262 PMC4703529

[B164] Panelos J. Massi D. (2009). Emerging role of Notch signaling in epidermal differentiation and skin cancer. Cancer Biol. Ther. 8 (21), 1986–1993. 10.4161/cbt.8.21.9921 19783903

[B165] Panelos J. Tarantini F. Paglierani M. Di Serio C. Maio V. Pellerito S. (2008). Photoexposition discriminates Notch 1 expression in human cutaneous squamous cell carcinoma. Mod. Pathol. 21 (3), 316–325. 10.1038/modpathol.3801007 18192969

[B166] Parkes G. M. Niranjan M. (2019). Uncovering extensive post-translation regulation during human cell cycle progression by integrative multi-'omics analysis. BMC Bioinforma. 20 (1), 536. 10.1186/s12859-019-3150-5 PMC6820968 31664894

[B167] Pavitt G. D. (2005). eIF2B, a mediator of general and gene-specific translational control. Biochem. Soc. Trans. 33 (Pt 6), 1487–1492. 10.1042/BST20051487 16246152

[B168] Pelullo M. Quaranta R. Talora C. Checquolo S. Cialfi S. Felli M. P. (2014). Notch3/Jagged1 circuitry reinforces notch signaling and sustains T-ALL. Neoplasia 16 (12), 1007–1017. 10.1016/j.neo.2014.10.004 25499214 PMC4309263

[B169] Peng D. Fu M. Wang M. Wei Y. Wei X. (2022). Targeting TGF-β signal transduction for fibrosis and cancer therapy. Mol. Cancer 21 (1), 104. 10.1186/s12943-022-01569-x 35461253 PMC9033932

[B170] Perkins N. D. (2012). The diverse and complex roles of NF-κB subunits in cancer. Nat. Rev. Cancer 12 (2), 121–132. 10.1038/nrc3204 22257950

[B171] Perl K. Ushakov K. Pozniak Y. Yizhar-Barnea O. Bhonker Y. Shivatzki S. (2017). Reduced changes in protein compared to mRNA levels across non-proliferating tissues. BMC Genomics 18 (1), 305. 10.1186/s12864-017-3683-9 28420336 PMC5395847

[B172] Phan T. P. Boatwright C. A. Drown C. G. Skinner M. W. Strong M. A. Jordan P. W. (2022). Upstream open reading frames control PLK4 translation and centriole duplication in primordial germ cells. Genes Dev. 36 (11-12), 718–736. 10.1101/gad.349604.122 35772791 PMC9296005

[B173] Pierfelice T. J. Schreck K. C. Dang L. Asnaghi L. Gaiano N. Eberhart C. G. (2011). Notch3 activation promotes invasive glioma formation in a tissue site-specific manner. Cancer Res. 71 (3), 1115–1125. 10.1158/0008-5472.CAN-10-0690 21245095 PMC3076023

[B174] Plaks V. Kong N. Werb Z. (2015). The cancer stem cell niche: how essential is the niche in regulating stemness of tumor cells? Cell Stem Cell 16 (3), 225–238. 10.1016/j.stem.2015.02.015 25748930 PMC4355577

[B175] Popovic D. Vucic D. Dikic I. (2014). Ubiquitination in disease pathogenesis and treatment. Nat. Med. 20 (11), 1242–1253. 10.1038/nm.3739 25375928

[B176] Proweller A. Tu L. Lepore J. J. Cheng L. Lu M. M. Seykora J. (2006). Impaired notch signaling promotes *de novo* squamous cell carcinoma formation. Cancer Res. 66 (15), 7438–7444. 10.1158/0008-5472.CAN-06-0793 16885339

[B177] Qi R. An H. Yu Y. Zhang M. Liu S. Xu H. (2003). Notch1 signaling inhibits growth of human hepatocellular carcinoma through induction of cell cycle arrest and apoptosis. Cancer Res. 63 (23), 8323–8329. 14678992

[B178] Quail D. F. Joyce J. A. (2013). Microenvironmental regulation of tumor progression and metastasis. Nat. Med. 19 (11), 1423–1437. 10.1038/nm.3394 24202395 PMC3954707

[B179] Reedijk M. Odorcic S. Chang L. Zhang H. Miller N. McCready D. R. (2005). High-level coexpression of JAG1 and NOTCH1 is observed in human breast cancer and is associated with poor overall survival. Cancer Res. 65 (18), 8530–8537. 10.1158/0008-5472.CAN-05-1069 16166334

[B180] Riemann A. Schneider B. Ihling A. Nowak M. Sauvant C. Thews O. (2011). Acidic environment leads to ROS-induced MAPK signaling in cancer cells. PLoS One 6 (7), e22445. 10.1371/journal.pone.0022445 21818325 PMC3144229

[B181] Ross K. E. Zhang G. Akcora C. Lin Y. Fang B. Koomen J. (2023). Network models of protein phosphorylation, acetylation, and ubiquitination connect metabolic and cell signaling pathways in lung cancer. PLoS Comput. Biol. 19 (3), e1010690. 10.1371/journal.pcbi.1010690 36996232 PMC10089347

[B182] Sadikovic B. Al-Romaih K. Squire J. A. Zielenska M. (2008). Cause and consequences of genetic and epigenetic alterations in human cancer. Curr. Genomics 9 (6), 394–408. 10.2174/138920208785699580 19506729 PMC2691666

[B183] Sakaguchi M. Miyazaki M. Takaishi M. Sakaguchi Y. Makino E. Kataoka N. (2003). S100C/A11 is a key mediator of Ca(2+)-induced growth inhibition of human epidermal keratinocytes. J. Cell Biol. 163 (4), 825–835. 10.1083/jcb.200304017 14623863 PMC2173690

[B184] Sallusto F. Geginat J. Lanzavecchia A. (2004). Central memory and effector memory T cell subsets: function, generation, and maintenance. Annu. Rev. Immunol. 22, 745–763. 10.1146/annurev.immunol.22.012703.104702 15032595

[B185] Salmena L. Carracedo A. Pandolfi P. P. (2008). Tenets of PTEN tumor suppression. Cell 133 (3), 403–414. 10.1016/j.cell.2008.04.013 18455982

[B186] Sansal I. Sellers W. R. (2004). The biology and clinical relevance of the PTEN tumor suppressor pathway. J. Clin. Oncol. 22 (14), 2954–2963. 10.1200/JCO.2004.02.141 15254063

[B187] Santos C. R. Schulze A. (2012). Lipid metabolism in cancer. Febs J. 279 (15), 2610–2623. 10.1111/j.1742-4658.2012.08644.x 22621751

[B188] Saran U. Chandrasekaran B. Tyagi A. Shukla V. Singh A. Sharma A. K. (2023). A small molecule inhibitor of Notch1 modulates stemness and suppresses breast cancer cell growth. Front. Pharmacol. 14, 1150774. 10.3389/fphar.2023.1150774 36909163 PMC9998682

[B189] Schreiber R. D. Old L. J. Smyth M. J. (2011). Cancer immunoediting: integrating immunity's roles in cancer suppression and promotion. Science 331 (6024), 1565–1570. 10.1126/science.1203486 21436444

[B190] Schulte-Hermann R. Bursch W. Kraupp-Grasl B. Oberhammer F. Wagner A. (1992). Programmed cell death and its protective role with particular reference to apoptosis. Toxicol. Lett. 64-65, 569–574. Spec No. 10.1016/0378-4274(92)90233-a 1471210

[B191] Schwanhäusser B. Busse D. Li N. Dittmar G. Schuchhardt J. Wolf J. (2011). Global quantification of mammalian gene expression control. Nature 473 (7347), 337–342. 10.1038/nature10098 21593866

[B192] Semenza G. L. (2003). Targeting HIF-1 for cancer therapy. Nat. Rev. Cancer 3 (10), 721–732. 10.1038/nrc1187 13130303

[B193] Semenza G. L. (2010). Oxygen homeostasis. Wiley Interdiscip. Rev. Syst. Biol. Med. 2 (3), 336–361. 10.1002/wsbm.69 20836033

[B194] Shahrzad S. Bertrand K. Minhas K. Coomber B. L. (2007). Induction of DNA hypomethylation by tumor hypoxia. Epigenetics 2 (2), 119–125. 10.4161/epi.2.2.4613 17965619

[B195] Sherman M. Y. Goldberg A. L. (2001). Cellular defenses against unfolded proteins: a cell biologist thinks about neurodegenerative diseases. Neuron 29 (1), 15–32. 10.1016/s0896-6273(01)00177-5 11182078

[B196] Sherr C. J. McCormick F. (2002). The RB and p53 pathways in cancer. Cancer Cell 2 (2), 103–112. 10.1016/s1535-6108(02)00102-2 12204530

[B197] Shou J. Ross S. Koeppen H. de Sauvage F. J. Gao W. Q. (2001). Dynamics of notch expression during murine prostate development and tumorigenesis. Cancer Res. 61 (19), 7291–7297. 11585768

[B198] Shvedunova M. Akhtar A. (2022). Modulation of cellular processes by histone and non-histone protein acetylation. Nat. Rev. Mol. Cell Biol. 23 (5), 329–349. 10.1038/s41580-021-00441-y 35042977

[B199] Silva J. Fernandes R. Romão L. (2019). Translational regulation by upstream open reading frames and human diseases. Adv. Exp. Med. Biol. 1157, 99–116. 10.1007/978-3-030-19966-1_5 31342439

[B200] Singh V. Ram M. Kumar R. Prasad R. Roy B. K. Singh K. K. (2017). Phosphorylation: implications in cancer. Protein J. 36 (1), 1–6. 10.1007/s10930-017-9696-z 28108801

[B201] Smyth M. J. Hayakawa Y. Takeda K. Yagita H. (2002). New aspects of natural-killer-cell surveillance and therapy of cancer. Nat. Rev. Cancer 2 (11), 850–861. 10.1038/nrc928 12415255

[B202] Sonenberg N. Hinnebusch A. G. (2009). Regulation of translation initiation in eukaryotes: mechanisms and biological targets. Cell 136 (4), 731–745. 10.1016/j.cell.2009.01.042 19239892 PMC3610329

[B203] Stransky N. Egloff A. M. Tward A. D. Kostic A. D. Cibulskis K. Sivachenko A. (2011). The mutational landscape of head and neck squamous cell carcinoma. Science 333 (6046), 1157–1160. 10.1126/science.1208130 21798893 PMC3415217

[B204] Stratton M. R. Campbell P. J. Futreal P. A. (2009). The cancer genome. Nature 458 (7239), 719–724. 10.1038/nature07943 19360079 PMC2821689

[B205] Stylianou S. Clarke R. B. Brennan K. (2006). Aberrant activation of notch signaling in human breast cancer. Cancer Res. 66 (3), 1517–1525. 10.1158/0008-5472.CAN-05-3054 16452208

[B206] Sui C. Zhuang C. Sun D. Yang L. Zhang L. Song L. (2017). Notch1 regulates the JNK signaling pathway and increases apoptosis in hepatocellular carcinoma. Oncotarget 8 (28), 45837–45847. 10.18632/oncotarget.17434 28507277 PMC5542231

[B207] Swinnen J. V. Brusselmans K. Verhoeven G. (2006). Increased lipogenesis in cancer cells: new players, novel targets. Curr. Opin. Clin. Nutr. Metab. Care 9 (4), 358–365. 10.1097/01.mco.0000232894.28674.30 16778563

[B208] Talora C. Sgroi D. C. Crum C. P. Dotto G. P. (2002). Specific down-modulation of Notch1 signaling in cervical cancer cells is required for sustained HPV-E6/E7 expression and late steps of malignant transformation. Genes Dev. 16 (17), 2252–2263. 10.1101/gad.988902 12208848 PMC186663

[B209] Thorne J. L. Campbell M. J. Turner B. M. (2009). Transcription factors, chromatin and cancer. Int. J. Biochem. Cell Biol. 41 (1), 164–175. 10.1016/j.biocel.2008.08.029 18804550

[B210] Tian Q. Stepaniants S. B. Mao M. Weng L. Feetham M. C. Doyle M. J. (2004). Integrated genomic and proteomic analyses of gene expression in Mammalian cells. Mol. Cell Proteomics 3 (10), 960–969. 10.1074/mcp.M400055-MCP200 15238602

[B211] Tomczak K. Czerwińska P. Wiznerowicz M. (2015). The Cancer Genome Atlas (TCGA): an immeasurable source of knowledge. Contemp. Oncol. Pozn. 19 (1a), A68–A77. 10.5114/wo.2014.47136 25691825 PMC4322527

[B212] Topalian S. L. Drake C. G. Pardoll D. M. (2015). Immune checkpoint blockade: a common denominator approach to cancer therapy. Cancer Cell 27 (4), 450–461. 10.1016/j.ccell.2015.03.001 25858804 PMC4400238

[B213] Trapani J. A. Smyth M. J. (2002). Functional significance of the perforin/granzyme cell death pathway. Nat. Rev. Immunol. 2 (10), 735–747. 10.1038/nri911 12360212

[B214] Trapnell C. Pachter L. Salzberg S. L. (2009). TopHat: discovering splice junctions with RNA-Seq. Bioinformatics 25 (9), 1105–1111. 10.1093/bioinformatics/btp120 19289445 PMC2672628

[B215] Valastyan S. Weinberg R. A. (2011). Tumor metastasis: molecular insights and evolving paradigms. Cell 147 (2), 275–292. 10.1016/j.cell.2011.09.024 22000009 PMC3261217

[B216] Valdez J. M. Xin L. (2013). The dual nature of Notch in tissue homeostasis and carcinogenesis. Cell Cycle 12 (4), 541. 10.4161/cc.23671 23370389 PMC3594250

[B217] van Limpt V. Chan A. Schramm A. Eggert A. Versteeg R. (2005). Phox2B mutations and the Delta-Notch pathway in neuroblastoma. Cancer Lett. 228 (1-2), 59–63. 10.1016/j.canlet.2005.02.050 16084642

[B218] Vedin L. L. Lewandowski S. A. Parini P. Gustafsson J. A. Steffensen K. R. (2009). The oxysterol receptor LXR inhibits proliferation of human breast cancer cells. Carcinogenesis 30 (4), 575–579. 10.1093/carcin/bgp029 19168586

[B219] Viatour P. Ehmer U. Saddic L. A. Dorrell C. Andersen J. B. Lin C. (2011). Notch signaling inhibits hepatocellular carcinoma following inactivation of the RB pathway. J. Exp. Med. 208 (10), 1963–1976. 10.1084/jem.20110198 21875955 PMC3182062

[B220] Viel S. Marçais A. Guimaraes F. S. Loftus R. Rabilloud J. Grau M. (2016). TGF-β inhibits the activation and functions of NK cells by repressing the mTOR pathway. Sci. Signal 9 (415), ra19. 10.1126/scisignal.aad1884 26884601

[B221] Vogel C. Marcotte E. M. (2012). Insights into the regulation of protein abundance from proteomic and transcriptomic analyses. Nat. Rev. Genet. 13 (4), 227–232. 10.1038/nrg3185 22411467 PMC3654667

[B222] Vogelstein B. Kinzler K. W. (2004). Cancer genes and the pathways they control. Nat. Med. 10 (8), 789–799. 10.1038/nm1087 15286780

[B223] Vogelstein B. Lane D. Levine A. J. (2000). Surfing the p53 network. Nature 408 (6810), 307–310. 10.1038/35042675 11099028

[B224] Volcic M. Karl S. Baumann B. Salles D. Daniel P. Fulda S. (2012). NF-κB regulates DNA double-strand break repair in conjunction with BRCA1-CtIP complexes. Nucleic Acids Res. 40 (1), 181–195. 10.1093/nar/gkr687 21908405 PMC3245919

[B225] Wahl M. C. Will C. L. Lührmann R. (2009). The spliceosome: design principles of a dynamic RNP machine. Cell 136 (4), 701–718. 10.1016/j.cell.2009.02.009 19239890

[B226] Wang E. T. Sandberg R. Luo S. Khrebtukova I. Zhang L. Mayr C. (2008). Alternative isoform regulation in human tissue transcriptomes. Nature 456 (7221), 470–476. 10.1038/nature07509 18978772 PMC2593745

[B227] Wang N. Ranalletta M. Matsuura F. Peng F. Tall A. R. (2006). LXR-induced redistribution of ABCG1 to plasma membrane in macrophages enhances cholesterol mass efflux to HDL. Arterioscler. Thromb. Vasc. Biol. 26 (6), 1310–1316. 10.1161/01.ATV.0000218998.75963.02 16556852

[B228] Wang Q. Zhang W. Qi X. Li J. Liu Y. Li Q. (2023). The mechanism of liver X receptor regulates the balance of glycoFAsynthesis and cholesterol synthesis in clear cell renal cell carcinoma. Clin. Transl. Med. 13 (5), e1248. 10.1002/ctm2.1248 37138531 PMC10157264

[B229] Wang Y. Shi J. Chai K. Ying X. Zhou B. P. (2013). The role of Snail in EMT and tumorigenesis. Curr. Cancer Drug Targets 13 (9), 963–972. 10.2174/15680096113136660102 24168186 PMC4004763

[B230] Wang Z. Gerstein M. Snyder M. (2009). RNA-Seq: a revolutionary tool for transcriptomics. Nat. Rev. Genet. 10 (1), 57–63. 10.1038/nrg2484 19015660 PMC2949280

[B231] Watson J. A. Watson C. J. McCrohan A. M. Woodfine K. Tosetto M. McDaid J. (2009). Generation of an epigenetic signature by chronic hypoxia in prostate cells. Hum. Mol. Genet. 18 (19), 3594–3604. 10.1093/hmg/ddp307 19584087

[B232] Webb B. A. Chimenti M. Jacobson M. P. Barber D. L. (2011). Dysregulated pH: a perfect storm for cancer progression. Nat. Rev. Cancer 11 (9), 671–677. 10.1038/nrc3110 21833026

[B233] Weinstein J. N. Collisson E. A. Mills G. B. Shaw K. R. Ozenberger B. A. Ellrott K. (2013). The cancer genome Atlas pan-cancer analysis project. Nat. Genet. 45 (10), 1113–1120. 10.1038/ng.2764 24071849 PMC3919969

[B234] Weng A. P. Ferrando A. A. Lee W. Morris J. P. Silverman L. B. Sanchez-Irizarry C. (2004). Activating mutations of NOTCH1 in human T cell acute lymphoblastic leukemia. Science 306 (5694), 269–271. 10.1126/science.1102160 15472075

[B235] Wethmar K. (2014). The regulatory potential of upstream open reading frames in eukaryotic gene expression. Wiley Interdiscip. Rev. RNA 5 (6), 765–778. 10.1002/wrna.1245 24995549

[B236] Wigerup C. Påhlman S. Bexell D. (2016). Therapeutic targeting of hypoxia and hypoxia-inducible factors in cancer. Pharmacol. Ther. 164, 152–169. 10.1016/j.pharmthera.2016.04.009 27139518

[B237] Williams A. B. Schumacher B. (2016). p53 in the DNA-Damage-Repair Process. Cold Spring Harb. Perspect. Med. 6 (5), a026070. 10.1101/cshperspect.a026070 27048304 PMC4852800

[B238] Wilson W. R. Hay M. P. (2011). Targeting hypoxia in cancer therapy. Nat. Rev. Cancer 11 (6), 393–410. 10.1038/nrc3064 21606941

[B239] Wirth M. Jira D. Ott A. Piontek G. Pickhard A. (2018). High NOTCH1 mRNA expression is associated with better survival in HNSCC. Int. J. Mol. Sci. 19 (3), 830. 10.3390/ijms19030830 29533972 PMC5877691

[B240] Wrzesinski S. H. Wan Y. Y. Flavell R. A. (2007). Transforming growth factor-beta and the immune response: implications for anticancer therapy. Clin. Cancer Res. 13 (18 Pt 1), 5262–5270. 10.1158/1078-0432.CCR-07-1157 17875754

[B241] Wu G. Wang Q. Xu Y. Li J. Zhang H. Qi G. (2019). Targeting the transcription factor receptor LXR to treat clear cell renal cell carcinoma: agonist or inverse agonist? Cell Death Dis. 10 (6), 416. 10.1038/s41419-019-1654-6 31138790 PMC6538631

[B242] Xie Y. Liu C. Qin Y. Chen J. Fang J. (2019). Knockdown of IRE1ɑ suppresses metastatic potential of colon cancer cells through inhibiting FN1-Src/FAK-GTPases signaling. Int. J. Biochem. Cell Biol. 114, 105572. 10.1016/j.biocel.2019.105572 31326465

[B243] Xing Z. Y. Sun L. G. Guo W. J. (2015). Elevated expression of Notch-1 and EGFR induced apoptosis in glioblastoma multiforme patients. Clin. Neurol. Neurosurg. 131, 54–58. 10.1016/j.clineuro.2015.01.018 25704190

[B244] Xu J. Yang X. Deng Q. Yang C. Wang D. Jiang G. (2021). TEM8 marks neovasculogenic tumor-initiating cells in triple-negative breast cancer. Nat. Commun. 12 (1), 4413. 10.1038/s41467-021-24703-7 34285210 PMC8292527

[B245] Xu L. Zhu Y. Xu J. Wu K. Li J. Xu W. (2012). Notch1 activation promotes renal cell carcinoma growth via PI3K/Akt signaling. Cancer Sci. 103 (7), 1253–1258. 10.1111/j.1349-7006.2012.02291.x 22463491 PMC7659298

[B246] Yan H. Qiu W. Koehne de Gonzalez A. K. Wei J. S. Tu M. Xi C. H. (2019). HHLA2 is a novel immune checkpoint protein in pancreatic ductal adenocarcinoma and predicts post-surgical survival. Cancer Lett. 442, 333–340. 10.1016/j.canlet.2018.11.007 30447255 PMC6357962

[B247] Yang L. Pang Y. Moses H. L. (2010). TGF-beta and immune cells: an important regulatory axis in the tumor microenvironment and progression. Trends Immunol. 31 (6), 220–227. 10.1016/j.it.2010.04.002 20538542 PMC2891151

[B248] Ye Y. Z. Zhang Z. H. Fan X. Y. Xu X. L. Chen M. L. Chang B. W. (2013). Notch3 overexpression associates with poor prognosis in human non-small-cell lung cancer. Med. Oncol. 30 (2), 595. 10.1007/s12032-013-0595-7 23645556

[B249] Yi L. Zhou X. Li T. Liu P. Hai L. Tong L. (2019). Notch1 signaling pathway promotes invasion, self-renewal and growth of glioma initiating cells via modulating chemokine system CXCL12/CXCR4. J. Exp. Clin. Cancer Res. 38 (1), 339. 10.1186/s13046-019-1319-4 31382985 PMC6683584

[B250] Zadra G. Photopoulos C. Loda M. (2013). The fat side of prostate cancer. Biochim. Biophys. Acta 1831 (10), 1518–1532. 10.1016/j.bbalip.2013.03.010 23562839 PMC3766375

[B251] Zage P. E. Nolo R. Fang W. Stewart J. Garcia-Manero G. Zweidler-McKay P. A. (2012). Notch pathway activation induces neuroblastoma tumor cell growth arrest. Pediatr. Blood Cancer 58 (5), 682–689. 10.1002/pbc.23202 21744479 PMC3264695

[B252] Zelcer N. Tontonoz P. (2006). Liver X receptors as integrators of metabolic and inflammatory signaling. J. Clin. Invest 116 (3), 607–614. 10.1172/JCI27883 16511593 PMC1386115

[B253] Zhang W. Jiang H. Zhang J. Zhang Y. Liu A. Zhao Y. (2014). Liver X receptor activation induces apoptosis of melanoma cell through caspase pathway. Cancer Cell Int. 14 (1), 16. 10.1186/1475-2867-14-16 24564864 PMC3941804

[B254] Zhao K. Wang Q. Wang Y. Huang K. Yang C. Li Y. (2017). EGFR/c-myc axis regulates TGFβ/Hippo/Notch pathway via epigenetic silencing miR-524 in gliomas. Cancer Lett. 406, 12–21. 10.1016/j.canlet.2017.07.022 28778566

[B255] Zhao M. Mishra L. Deng C. X. (2018). The role of TGF-β/SMAD4 signaling in cancer. Int. J. Biol. Sci. 14 (2), 111–123. 10.7150/ijbs.23230 29483830 PMC5821033

